# Connecting genomic islands across prokaryotic and phage genomes via protein families

**DOI:** 10.1038/s41598-023-27584-6

**Published:** 2023-01-07

**Authors:** Reem Aldaihani, Lenwood S. Heath

**Affiliations:** 1 Department of Computer Science, Kuwait University, Kuwait City, State of Kuwait; 2 Department of Computer Science, Virginia Tech, Blacksburg, VA USA

**Keywords:** Computational biology and bioinformatics, Bioinformatics, Genomic analysis

## Abstract

Prokaryotic genomes evolve via horizontal gene transfer (HGT), mutations, and rearrangements. A noteworthy part of the HGT process is facilitated by genomic islands (GIs). While previous computational biology research has focused on developing tools to detect GIs in prokaryotic genomes, there has been little research investigating GI patterns and biological connections across species. We have pursued the novel idea of connecting GIs across prokaryotic and phage genomes via patterns of protein families. Such patterns are sequences of protein families frequently present in the genomes of multiple species. We combined the large data set from the IslandViewer4 database with protein families from Pfam while implementing a comprehensive strategy to identify patterns making use of HMMER, BLAST, and MUSCLE. we also implemented Python programs that link the analysis into a single pipeline. Research results demonstrated that related GIs often exist in species that are evolutionarily unrelated and in multiple bacterial phyla. Analysis of the discovered patterns led to the identification of biological connections among prokaryotes and phages. These connections suggest broad HGT connections across the bacterial kingdom and its associated phages. The discovered patterns and connections could provide the basis for additional analysis on HGT breadth and the patterns in pathogenic GIs.

## Introduction

Prokaryotes are among the most diverse organisms on planet Earth in evolutionary biology and also for any discipline considering prokaryotic genomes^1^. They have the ability to live and proliferate in numerous diverse environments while exchanging their genetic material horizontally using Horizontal Gene Transfer (HGT)^2^. HGT commonly takes place between bacteriophages (phage) and their prokaryotic hosts. A phage is a type of virus that infects and replicates within prokaryotes.

By HGT a genome may obtain a sequence sourced from other microorganisms; this sequence is called a genomic island (GI) and typically contains several genes that have evidence of independent origins and play a central role in prokaryotic evolution. GIs contain several features that assist in their identification from the remainder of a bacterial genome based on their structure ^3^. One of the primary characteristics of GIs is that their phyletic patterns are different from the host genome structure because of the sporadic distribution of GIs. The interest in identifying prokaryotic GIs has recently increased, which is evident judging by the various computational tools developed most of them to predict GIs ^4^. Furthermore, researchers have focused on introducing GIs’ features, however, not every feature is required in a region for that region to be classified as a GI^5–7^. Moreover, Hsiao^8^ analyzed prokaryotic pathogenicity GIs and investigated their computational characterization, while bioinformatics studies have demonstrated that novel genes appear more in GIs^9^. Even though numerous computational methods have been devised for the detection of different kinds of GIs and their features, researchers are yet to deeply investigate the GI patterns or HGT connections between species in terms of GIs.

The main contribution of this paper is a pipeline whose objective is to extract patterns from GIs and use them in connecting GIs across prokaryotic and phage genomes via protein families. Regarding the patterns, the aim is to show that there patterns usually present in prokaryotes, even when distantly related. These patterns consist of specific protein families, meaning their proteins are usually located on several GIs together in a particular order. Regarding the connections, which defined as an HGT relation resulting from an interaction between a phage and a number of prokaryotic species, these are formed using the discovered patterns. There is a solid historical relationship regarding the evolution between prokaryotes and phages. However, so far, the role of GIs in this phylogenetic relationship has not yet been studied intensely. Research analysis has showed that HGT could occur between prokaryotes even if they are distantly related. This may be due to several reasons, including their presence in the same environment and the phages playing a significant role in this process. Biologically, the patterns and connections can be used as a foundation for research problems related to the prokaryotic and phage communities.

## Materials and methods

### Data set

The prokaryotic data set of GIs used in this research was retrieved from the IslandViewer4 website in April, 2020. The GIs in IslandViewer4 are predicted by four different tools; SIGI-HMM, IslandPick, Islander, and IslandPath-DIMOB^10^. The data set in this study contained 13,897 prokaryotic genomes, where the number of GIs totals to 384,162 and the total number of protein sequences in these GIs is 4,725,173. The phage data set used in this research was downloaded from the NCBI website. Furthermore, the Pfam database was used along with HMMER to generate the protein families of protein sequences ^11^.

### Problem statement

**Figure 1:**
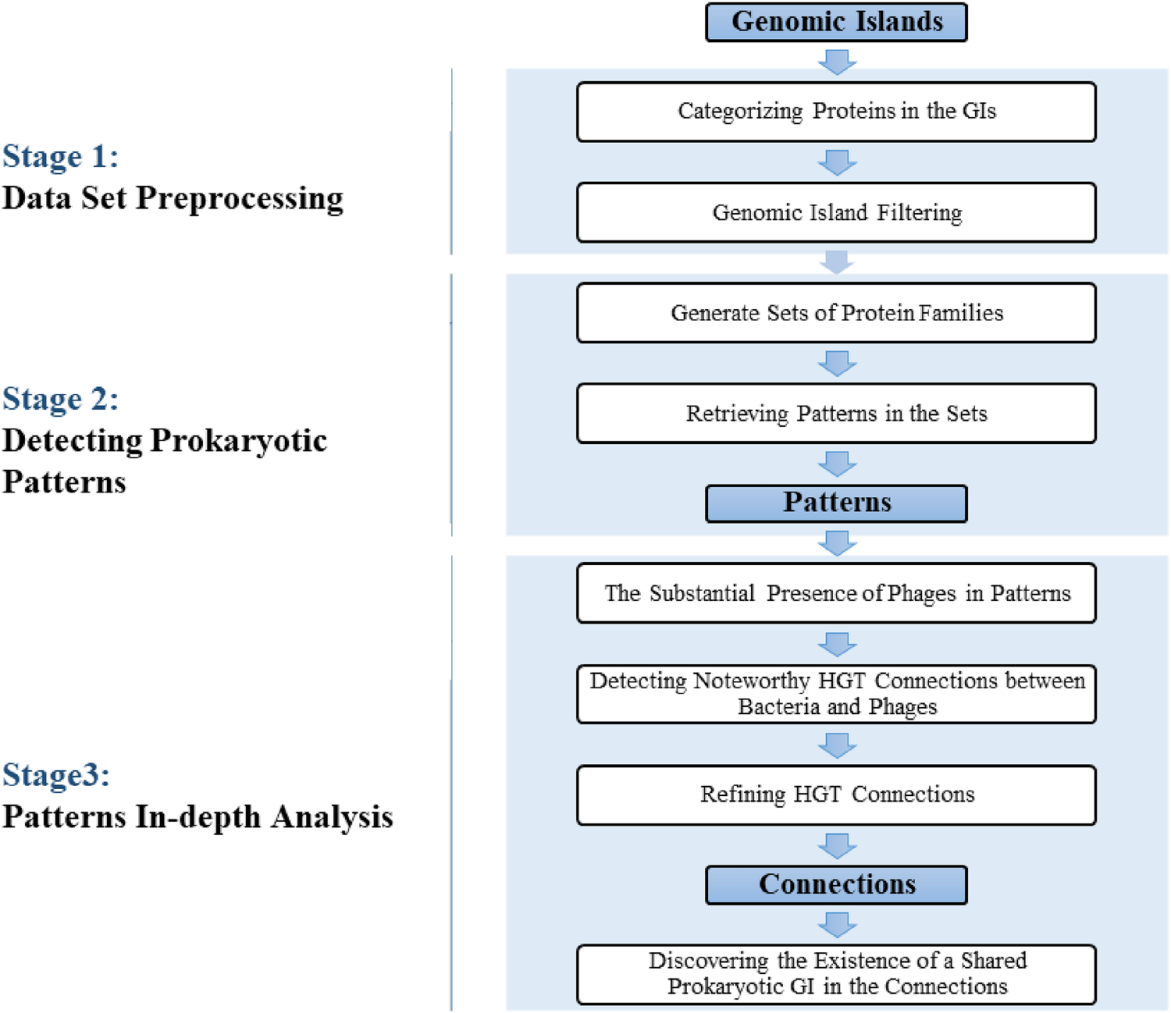
The pattern-connection pipeline is divided into three main stages. The first stage is the data set preprocessing. The function of this stage is to generate protein families of the proteins in the GIs and filter the GIs. The second stage is to obtain the patterns by generating sets of protein families using the Apriori algorithm before retrieving patterns from the sets and filtering them to get the promising patterns. The third stage of the pipeline is related to the connections. In this part, the patterns are as input and go through three major steps to get the connections. In these three steps BLAST is used to detect matches for the prokaryotic GIs with the phage database to obtain connections and then filter them to obtain the promising ones.

We formulated our research problem mathematically. The prokaryotic data set contains a number, *N*, of prokaryotic genomes ($\mathscr{G}$) from numerous prokaryotic species; $\mathscr {G}= \{G_1, G_2, G_3, \dots , G_N\}$ The webpage for a genome $G_i$ contains the genome sequence, the GI sequences in the genome, the protein sequences in each GI, and other information related to the genome and the GIs. As the main idea of the research is based on protein families, the research input is the proteins within each GI. Each GI contains a sequence of genes, in order, which give rise to a sequence of proteins, say, $P_1, P_2, P_3, \dots , P_K$ where *K* is a characteristic of the GI. We analyzed the entirety of the GIs through several steps illustrated in the pipeline in Fig. 1. The pseudocode for all the pipeline algorithms can be found in the supplemental document.

The first stage of the pipeline is to retrieve the GI proteins from IslandViewer4 and map them from the set of proteins to the set of protein families; $f(P_1), f(P_2), f(P_3), \dots , f(P_K)$, using hmmsearch and Pfam and then filter the GIs. In stage two, the first step is to identify the most frequent sets *T* of protein families in the universe of GIs; $\mathscr {S} = \{S_1, S_2, \dots , S_T\}$. One set $S_j\in \mathscr {S}$ contains is a number *Z* of protein families that frequently exist in some of the GIs, though not necessarily in the same order or even consecutively. The second step is to retrieve patterns in these sets and filter them under certain conditions to obtain promising patterns using BLAST and the new algorithms. A pattern is a number of protein families that frequently exist in the GIs in the same order that they present themselves in the pattern, but not necessarily consecutively. In stage three , we took a deeper look at the resulting patterns to connect GIs across prokaryotic and phage genomes via protein families and this stage results in a number of connections; $C_1, C_2, \dots , C_L$. A connection *C* is a triple consisting of a pattern *P*, a phage genome *H*, and a set of bacteria species; $C = (P, H, {\{B_1, B_2, \dots , B_d \}})$. The phage and bacterial species share the common pattern *P* in their genomes and the number *d* of bacteria will vary.

### Analytical flow

This section explains in detail the three stages of the Pattern-Connection pipeline in Fig. 1.

#### Data set preprocessing

The GIs in the data set are from numerous prokaryotic species that are from various taxonomies. However, it was noticed that there were more GIs in certain species than in other species within the data set, which is demonstrated in the *Top species in the data set* table in the supplemental document, with the five most frequent species that the GIs in the data set belong to. Regarding the phylogenetic relation between the top species, from a taxonomy point of view, all the species were from the same phylum known as Proteobacteria. It was noticed that the five most common bacteria species were common in the rod shape (i.e. bacillus) which could means GIs can usually exist in bacillus bacteria. In this stage of the pipeline, the focus is on generating the protein families of the protein sequences in the prokaryotic GIs to use them in the next stage to detect the patterns. The identification of the protein family of each protein sequence was performed using hmmsearch and pfam ^11^. After running hmmsearch, the result came to 3,071,081 protein sequences assigned to families. This number is derived from the lowest E value condition and the Ribosomal protein families condition explained in the *Ribosomal Protein Families* section in the supplemental document. The total number of GIs after applying the latter condition is 368,339.

#### Detecting prokaryotic patterns

In stage two of the pipeline, the strategy for detecting prokaryotic patterns is presented.

- Generate Sets of Protein Families: The first step of generating the patterns was performed by generating the sets of protein families using the Apriori algorithm ^12^. The protein families of the protein sequences were chosen for two reasons. The first reason is that they are more general than proteins and there is a high possibility to get patterns in many GIs. The second reason is based on one of the research aims, which is to detect the structure of the GIs which is mainly related to the function of the proteins in the GIs, since there could be two different proteins that give the same function. Therefore, dealing with the protein families of the protein sequences was chosen as it achieves what is desired in this research.
- Retrieving Patterns in the Sets: In this step, the aim is to obtain patterns from the sets. This is performed by generate all the possible patterns for each set. Then, the promising patterns are selected under certain conditions. Obtaining the promising patterns is performed using the *Patterns* algorithm. There are a number of conditions applied to the patterns to obtain the wanted patterns. First, patterns that existed in fewer than ten GIs were removed from the study. Second, patterns size have to be between 3 and 5 protein families. This range has been chosen since it was observed that when patterns become large in size, they are biased towards a particular species, and when the pattern is very small, like a pattern with two protein families, they exist in a vast number of GIs due to them often being very common as well as there not being anything special about them most of the time. The third condition is a taxonomy condition which is the GIs have to be exists in species from the most frequent seven orders in the data set. The most frequent orders were used as a condition for filtering the patterns because most of the GIs in the data set belong to these orders. With this condition, we are aiming for GIs that exist in species that are common to have GIs in their genomes, which means deal with more reliable GIs. The analysis showed that the number of orders less than seven returns a large number of patterns (i.e., Hundreds). Here we aim to select the most promising patterns. Furthermore, using the number of orders greater than seven did not retrieve any pattern. It has been noticed that the most frequent protein families in the patterns are the phage protein families, which proves that these patterns could have originated from phages.

#### Patterns in-depth analysis

In stage three of the pipeline, the patterns went through intensive analysis, leading to several findings.

- The Substantial Presence of Phages in Patterns: As mentioned previously, there are indications that the origin of the GIs possibly came from phages since in the GIs structure there are proteins that belong to phage protein families. The *Presence of Phages in the Patterns* algorithm is composed of a number of steps that lead to detecting if these patterns exist in phages. The algorithm retrieves the GIs that contain this pattern in their structure, which is performed in the same pattern order, then after collecting the GIs that have this pattern, the algorithm calls BLASTp (-evalue=10e-10) for each GI proteins against the viral protein database in order to find matches.
- Detecting Noteworthy HGT connections between Bacteria and Phages: In this step , there have been two algorithms implemented to generate the HGT connections. The first is the *Patterns (Phage Information)* algorithm that retrieves the successful GIs, which are the GIs whose proteins all had a viral match. Later, the GIs’ proteins are parsed to generate the genome accession number of the viral proteins using the EFetch function in the Entrez package. The aim of this step is to detect a viral genome accession that has a number of proteins that match the same number of bacteria proteins in the GI. These viral genome accessions were reported considering the largest E value among all the proteins in the GI. The algorithm results in connections where each one contained a GI and a viral genome that matched the GI with the pattern as well as the E value, which shows the quality of this match. A viral genome could match with more than one GI, therefore, the *Extracting HGT Connections* algorithm has been used to list all of the viral genomes and for each viral genome to list all the matched bacteria GIs along with their E values and the pattern that exists in the viral genomes and GIs. This is performed because a deep analysis is performed on phage and the related GIs with an aim to detect the most interesting Phage-Bacteria matches.
- Refining HGT Connections: Considering the previous step, a viral genome and a group of all bacteria GIs represents a possible HGT connection between them. This group of bacteria could be closely related or distantly related, where the distantly related bacteria could be related in terms of genome content and not yet discovered. The objective of this research is to detect the most interesting phages that infect bacteria that are from different lineages. This is performed by using the *Filtering HGT Connections [Taxonomy]* algorithm, where for each connection the algorithm obtains the common lineage of the bacteria then take the connections with a lineage length of less than five, which means the bacteria could be from the same order or higher. Another condition is related to the E value that aims to keep the bacteria with small E values (i.e., smaller than 10e-100) because bacteria with small E values could have more evidence of HGT with the phage. The next step is to dive deep into each connection by studying the pattern proteins in each species in the connection by using the *Connections* algorithm to get the promising connections. This is performed by retrieving the phage and bacteria proteins (i.e., Pattern proteins) then using BLAST, where the query sequence is the phage proteins and the subject sequence is the bacteria GI proteins. The scientific name of the BLAST result should be the same specific name as the bacteria GI that has this protein provided in the connection to maintain this phage-bacterium GI connection, otherwise, the protein is eliminated. Furthermore, the percent identity value should be greater than or equal to 85%, as the higher the percent identity is, the more significant the match is. Also, the phage and bacteria should be unrelated, which means the phage should not be a well-known phage that already infects the bacteria. Another investigation was also performed between the group of bacteria on the same connection. In this case, the proteins for each two bacteria are BLASTed sequentially and in reverse to make sure that the resulting scientific name is the same as the bacteria that hold the two GIs containing the two proteins. All of the species in the same connection are studied together because the GIs have evidence of horizontal gene transfer, is already published^10^. Therefore, the connection that we have is also evidence that there is a high possibility of HGT between these species.
- Discovering the Existence of a Shared Prokaryotic GI in the connections: An analysis using the *Connections (Extract Species Subsequences)* algorithm is performed to study the connection species to understand what they have in common by analyzing their genomes using BLAST, and discovering the possibility of the existence of a shared GI between the species. Furthermore, the next step of the algorithm is to use MUSCLE, a multiple sequence alignment tool, to investigate the similarity between the discovered GIs in the same connection.

## Results

In this section, the results of the protein families sets, patterns, and connections were illustrated.

### Protein families sets

This stage shows the results of the most frequent sets of protein families in the GIs that were generated using the Apriori algorithm with a support value equating to 0.006. This value is chosen according to system space capabilities and it was noticed from the results that when a set gets larger (i.e., the support value decreases), the number of species that have this set decreases, which could lead to a specific species which is the most frequent species in the data set, such as E.coli. This conflicts with the target of the research, which is to detect patterns that exist in numerous species. According to this support value, there were 640 sets of protein families in GIs with sizes between 2 and 7 protein families. More information regarding the resulting sets exists in the supplemental document in the *Sets of Protein Families* section.

**Figure 2:**
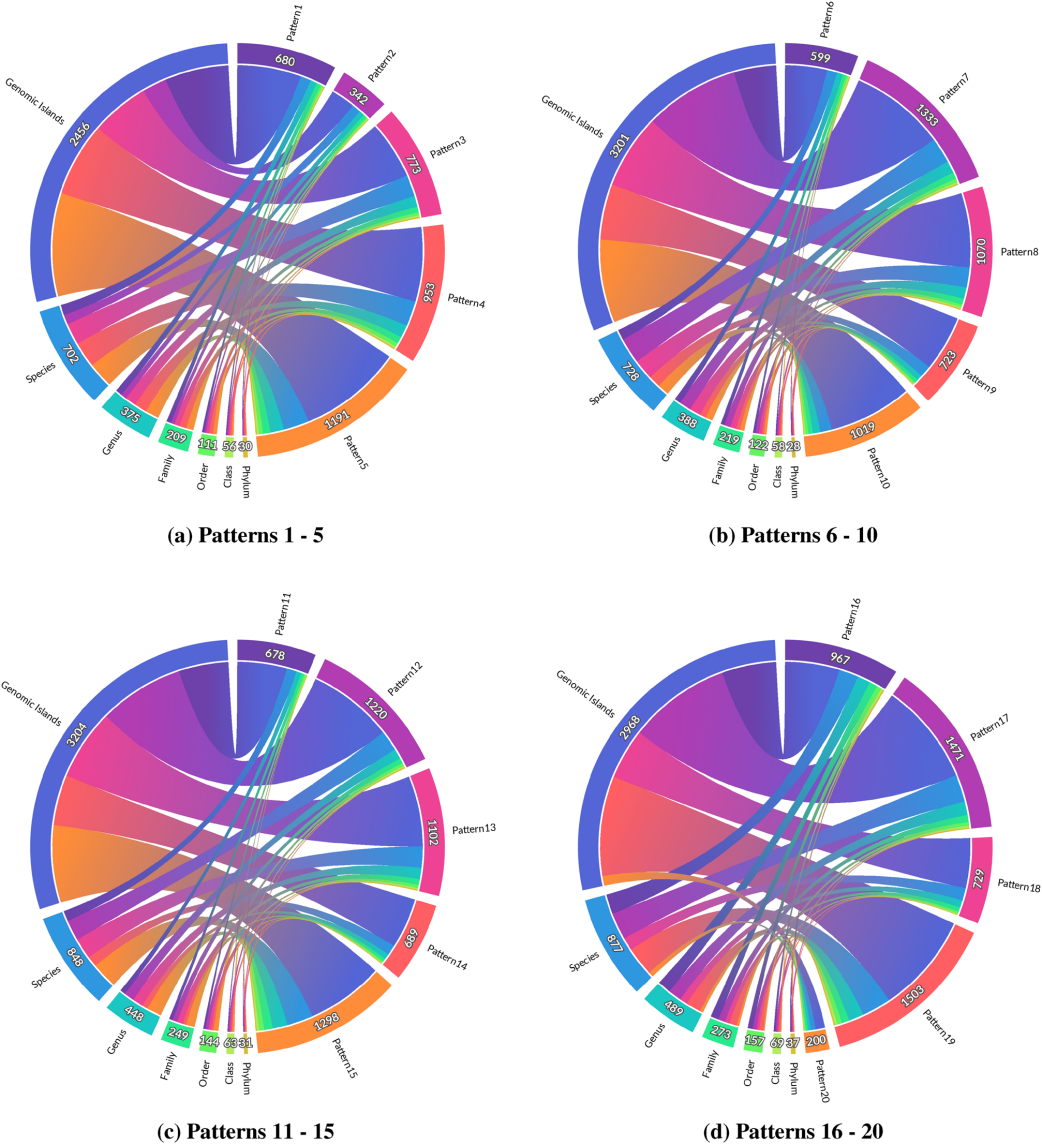
Chord diagrams show information about the taxonomy levels for each pattern. For each pattern, the diagram illustrates the number of GIs that have the pattern in their structure and the number of species, genera, families, orders, classes, and phyla in these GIs.

### Patterns

The initial resulting patterns at this part of the analysis was 33,448 patterns from the 640 original sets. The *Patterns* algorithm filters the resulting patterns to get the most promising ones that equated to 20 that are from size three except for the last one, which is of size four, as shown in the *Patterns* table in the supplemental document. The table shows that phage protein families exist in almost all the patterns, which proves that these patterns could have originated from phages. Furthermore, it shows that the most frequent protein families in the patterns are HNH, Phage_capsid, Terminase_1, and Phage_portal. The HNH protein family exists in many phages, and its proteins’ location in the phage genome is next to the terminase proteins, which is highly conserved^13^. There are many studies that have shown that the presence of HNH and terminase proteins together is essential for phage activities^14^ and this could prove that they have a significant role in the HGT process. Regarding Phage_capsid and Phage_portal protein families, they both play an essential role during the phage infection process. The table shows that when these protein families exist in a particular pattern, they are present in different arrangements, as shown in the colored rows. In general, the presence of these four protein families in the patterns may indicate that these are the essential protein families that lead to the success of the HGT process.

**Figure 3:**
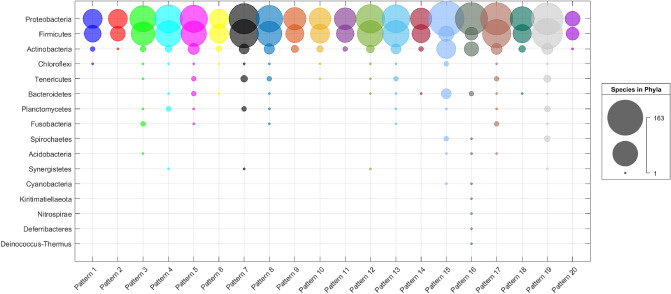
The number of the phylum’s species in each pattern. The bubble chart illustrates the resulting patterns in more detail, where there are all the patterns in the x-axis and the phylum names in the y-axis. A bubble for a pattern *P* and phylum *Phy* represents the number of species that belong to phylum *Phy* and have the pattern *P* in their structure.

In regards to taxonomy, Fig. 2 shows chord diagrams of the taxonomy levels for each pattern. Each chord diagram shows the taxonomy information for a number of the patterns. Overall, the figure shows that these patterns are diverse since they exist in numerous taxonomy levels and this could indicate that these patterns may strongly represent an essential part of the GIs. The bubble chart on Fig. 3, illustrates the resulting patterns in more detail. It is evident from the chart that all the patterns exist in species from the Firmicutes, Proteobacteria, and Actinobacteria phyla. The presence of the Proteobacteria phylum is expected because most of the GIs are from species that belong to the Proteobacteria phylum. Furthermore, the Proteobacteria phylum includes various pathogens, such as Escherichia and Salmonella, which play a significant role in the HGT process in prokaryotic. The presence of Firmicutes and Actinobacteria in all patterns may indicate that their GIs have a structure similar to the Proteobacteria GIs structure. This proves that there is a specific structure of GIs in prokaryotic. It is worth mentioning that Firmicutes and Actinobacteria bacteria can be found in various environments, and they include some pathogens and they can survive extreme conditions ^15^. Figure 3 shows that pattern number sixteen (Phage_integrase, HTH_Tnp_1, rve) exists in many GIs that are from eleven phyla, and pattern number nineteen exists in many GIs that are from ten different phyla. These two patterns could be essential in GIs since they exist in numerous phyla. The pattern (Phage_integrase, HTH_Tnp_1, rve) is explained in detail in the *The “Phage_integrase, HTH_Tnp_1, rve” Pattern* section in the supplemental document. Instead of demonstrating every single pattern in detail, this one example serves to demonstrate that, like in all other patterns, a pattern contains species that have differences on many points. In general, the Figs. 2 and 3 show that the patterns can be found in various species and each pattern is present in at least three different phyla of bacteria.

### Connections

A connection reflects a HGT relation between a phage and a number of prokaryotic species. The *Connections* table in the supplemental document contains the information on the resulting connections.

**Figure 4:**
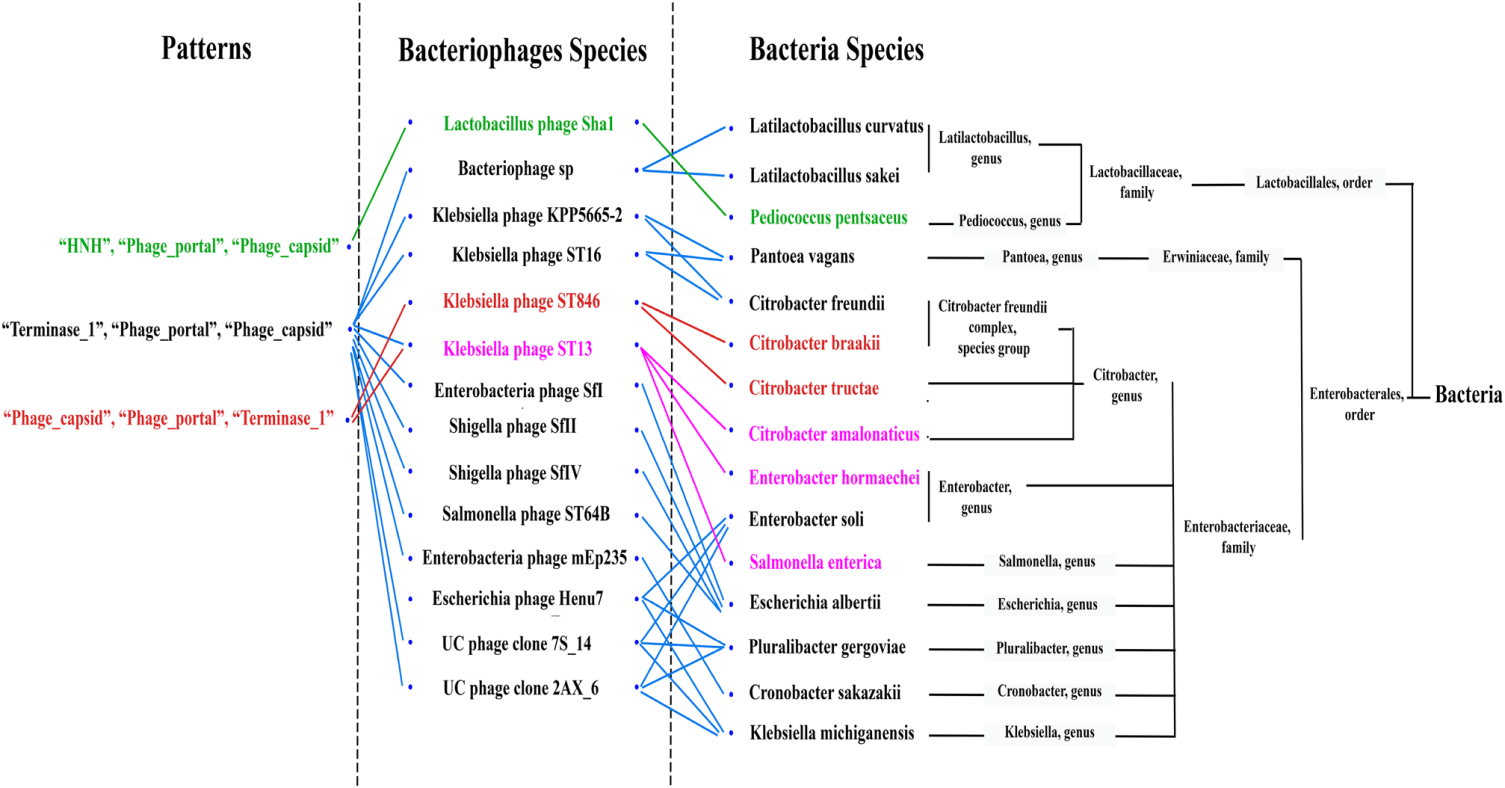
A tripartite graph of the connections. Going from left to right, the first set represents the patterns, the next set represents the phage species, and the following set shows the bacteria species. A phylogenetic tree of the bacteria species is added on the right side that represents the taxonomy relation between the bacteria. In the patterns part, each pattern has a color and a colored link between any two sets shows that these species have the same pattern. For example, the red pattern exists in one phage, which means one connection that is composed of one phage and two bacteria that are from different species.

Figure 4, shows the *Connections* table content as a tripartite graph with three independent sets representing the three main parts of each connection. Formally, let $Tri\_G$=($Tri\_P$, $Tri\_Ph$, $Tri\_B$, $E\_PPh$, $E\_PhB$) be a tripartite graph with the following sets:

$$\begin{aligned} Tri\_P= & {} \{tri\_p1, tri\_p2, tri\_p3\} \\ Tri\_Ph= & {} \{tri\_ph, \dots ,tri\_ph14\}\\ Tri\_B= & {} \{tri\_b1, \dots ,tri\_b15\} \\ \end{aligned}$$

The $Tri\_P$ set represents the patterns, next comes the phage species set ($Tri\_Ph$), then the bacteria species set ($Tri\_B$). The edges between the sets are represented as $E\_PPh$ and $E\_PhB$, where $E\_PPh$ represents the edges between the patterns and the phages, while $E\_PhB$ represents the edges between the phages and bacteria. A connection in the graph is represented as an edge between the pattern set and the phage set as well as another edge(s) between the phage set and bacteria set. As shown in the phylogenetic tree of bacteria species, for some connections, their bacteria are distantly related. For instance, in the pattern (Pattern: “Terminase_1”, “Phage_portal”, “Phage_capsid”.) the bacteria *Pantoea vagans* and *Citrobacter freundii* are from two different families but in the same connection with *Klebsiella phage* KPP5665-2. In this research, there are many connections, as shown in Fig. 4,where a lot of exciting information can be seen in each one. A connections that deserves research and scrutiny is one that consists of two compounded connections where the phage named *Klebsiella phage* ST13-OXA48phi12.2 can be found, colored in pink as a result of the two patterns blue and red being combined. The *Klebsiella phage* and bacteria in this connection share two patterns in their genome. However, despite the bacteria species belonging to the same bacteria family, they are from different genera. Detailed information about the bacteria species in this connection is explained in the *Compound connection* section in the supplemental document. Overall, in this connection, the bacteria species share the fact that they are usually present in the same locations, such as intestines or the intestinal tract of humans ^16–21^. It is important to mention that these bacteria have a serious effect on many kinds of living organisms including humans and various kinds of animals. The bacteria species may have a strong relation, which deserves to be studied in depth and may assist in answering questions about specific diseases related to them since these bacteria are in the same connection with a phage.

An analysis performed on all the phages in the connections is presented in the *Analysis of the Phages in the connections* section in the supplemental document.

### The existence of a shared prokaryotic GI in the connection species

In this step, the analysis aims to investigate more about the connections by analyzing the genomes. The *Connections (Phages and Bacteria BLAST Analysis)* table in the supplemental document shows the BLAST result between the genomes of the species within each connection. The E value for all the BLAST results is zero, therefore, it is not included in the table. The identity values in general are around the 80’s and 90’s, however, there are few 70’s cases. The *Connections (GIs Coordinates Information)* table in the supplemental document also shows the BLAST between the genomes for each connection with more information regarding the alignment included. *Connections (GIs Coordinates Information)* table shows that the resulting alignment sequence between the phage and the bacteria genome has almost the same coordinates as those of the GI in the bacteria genome on the IslandViewer4 website. In more details, the analysis shows that for each connection *C*, when BLASTing the phage genome *H* against the genome of bacterium $B_z$, the resulting alignment (i.e. subsequence) coordinates are similar to the GI coordinates in the bacterium $B_z$. Moreover, for a connection *C* the resulting phage *H* coordinates against the bacteria ${\{B_1, B_2, \dots , B_d \}}$ in most of the cases is almost the same coordinates. Therefore, from the aforementioned two observations, an assumption is created, which is that the resulting subsequences between the phage *H* and the bacteria ${\{B_1, B_2, \dots , B_d \}}$ are actually only one GI that is common between them. This means the genomes in the same connection *C* share the same GI, but the GI is not identical in all genomes in the same connection because the GI has been changed over time, which could be due to the mutations and movement of the GI between organisms. The MUSCLE result shows a very good alignment between the sequences in each connection. This is evidence that the sequences in the connection are the same sequence, which is the GI. The phylogenetic trees of the connections that composed of more than one bacteria species are presented in the supplemental document in the *MUSCLE phylogenetic trees* section.

## Discussion

There are many GI prediction tools, but most of them are to detect GIs. Therefore, we have endeavored to take a new direction to further understand GI’s. This research aims to study the structures of GIs in-depth to extract patterns from them that were used to obtain biological connections between prokaryotes and phages. In this study, protein families were used as the basic unit for the research instead of proteins as they are more informative. The analysis on protein families showed that most GIs contain three protein families in their structure, which may indicate that there are three essential components or protein functions in GIs. Moreover, the analysis showed that most GIs exist in rod shape species and this may indicate a possible relation between the HGT and the rod shape A possible factor could be the surface area to volume ratio as the rod shape gives a broad surface area per unit volume, which could assist in facilitating the attachment to bacteria and subsequently help to transfer the genetic material^22^. The discovered patterns show that the GIs have a specific structure that makes them sub-sequences of particular genes that could have a crucial role in changing the biological function of the species. Patterns are found in bacteria species that are closely related and distantly related, meaning that the HGT frequently happens across a substantial part of the bacterial communities. All patterns exist in species that differ in phyla, and in each pattern, at least three species are from different bacteria phyla. It has also been observed that most of the resulting patterns contain phage protein families, which is a significant sign that these GIs could originate highly from phages. In the in-depth analysis, the resulting connections consist of a phage, a group of bacteria, and a common pattern between them. The analysis showed that the species in the connection are similar in genome content despite being distantly related. Furthermore, the common pattern between the species assists in discovering a GI that is common between them, which is the same as the GI that is provided in IslandViewer4. Therefore, species in the same connection have a very high probability of HGT. It is essential to know that these connections were reached through a large number of filters that led to the deletion of a large number of species to achieve a solid biological connection. A deep analysis of the connections could assist in understanding the relationship between bacteria species and the phage as well as the relationship between the bacteria together, and this overall could answer many biological questions. For example, for the compound connection in the results section, the investigation showed that the bacteria species could have severe effects (i.e., diseases) on humans and animals. Therefore, an analysis could assist in understanding the cause of the diseases from these bacterial species and the phage and possibly finding the proper solution. As future work, the common GI in the HGT connections could be studied further to bring to light the origin and direction of the GI in each connection.

## Supplementary Information

Supplementary Information.

## Data Availability

The prokaryotic GIs data set analysed during the current study is available on the IslandViewer website: https://www.pathogenomics.sfu.ca/islandviewer/. All the Python codes that were used in this research are available in the GitHub repository: https://github.com/Reemcsvt/Prokaryotic-genomic-island-structure. Also, the genome accession number list, where the GIs are located, is available in the *Genomes_accession_number_list* file on the same GitHub repository.
