# Supplementary material for: Connecting genomic islands across prokaryotic and phage genomes via protein families
Source: Sci Rep. 2023 Jan 7;13:344. doi: 10.1038/s41598-023-27584-6 (PMC9825383; doi:10.1038/s41598-023-27584-6)
Supplement: Supplementary file 1 — Supplementary Information. [file 41598_2023_27584_MOESM1_ESM.pdf]

Supplemental Document for Connecting Genomic Islands across Prokaryotic and Phage Genomes via Protein Families

Reem Aldaihani
Department of Computer Science
Virginia Tech
Blacksburg, VA

Lenwood S. Heath
Department of Computer Science
Virginia Tech
Blacksburg, VA

December 29, 2022

Contents

A Ribosomal Protein Families **2**

B A Deeper look at Protein Families **2**

C Sets of Protein Families **8**

D The "Phage_integrase, HTH_Tnp_1, rve" Pattern **9**

E Compound connection **12**

F Analysis of the Phages in the connections **13**

G Supplemental Algorithms **14**

H Supplemental Tables **22**

I MUSCLE Phylogenetic Trees **27**

A Ribosomal Protein Families

In this section, an analysis was performed to analyze the prokaryotic GIs to make an overview of the data set and how it would contribute to the research. GIs were treated as sets of protein families and a clustering algorithm (i.e., BiMax algorithm [37]) was used to cluster these sets. There were notable sets in some clusters with almost the same content that is Ribosomal protein families but in different orders. It was noticed that within each species, there was one GI that contained all of these protein families and this GI was predicted by the IslandPath-DIMOB tool for the most part. Therefore, based on the gene annotations, this looks like a ribosomal protein operon, so, in theory, this could be present in all cellular life. It is possible that this part of the genome had been incorrectly predicted as a GI on one or a few genomes, and the genes that come from this part of the genome are not horizontally transferred to the host genome, which means that they should not be part of a GI. GIs will frequently have a sequence composition that is considerably dissimilar compared to the host's genome due to the different genome sequence compositions found in various bacterial lineages. Genomic island predictors that apply the sequence composition technique are always heavily dependent on this fact. IslandPath-DIMOB predicts GIs by using the dinucleotide sequence composition bias technique along with the presence of mobility genes. Therefore, GIs containing more than 50% of ribosomal protein families were eliminated from the data set used in the research.

B A Deeper look at Protein Families

In this section, the protein families in the data set were analyzed. The GIs in the data set are composed of protein sequences and these protein sequences belong to protein families from 9,121 different protein families.

Figure S(1), shows that the GIs in the data set have protein sequences that belong to protein families ranging from one protein family to 194 protein families.

This means the GIs in their structure have a number of protein families ranges from one protein family to 194 protein families. Furthermore, the figure shows that most of the GIs in the data set have a number of protein families ranging from 1 to 9 in their structure. It is worth mentioning that most of the GIs, which equates to 51883 GIs in the data set, have three protein families in their structure. Moreover, there is an outlier GI in the data set that has 947 protein families and this GI belongs to the Lactobacillus curvatus KG6 strain (NZ_CP022475.1). Lactobacillus curvatus KG6 demonstrated phenotypic novobiocin resistance when it was isolated on de Man-Rogosa-Sharpe agar from a fermented meat product similar to salami that was bought from a retailer in Switzerland in 1999 [21].

In general, it has been noticed in the data set that the majority of bacteria genera that have species containing GIs in the range between 58 and 194 are Salmonella, Escherichia, and Bacillus

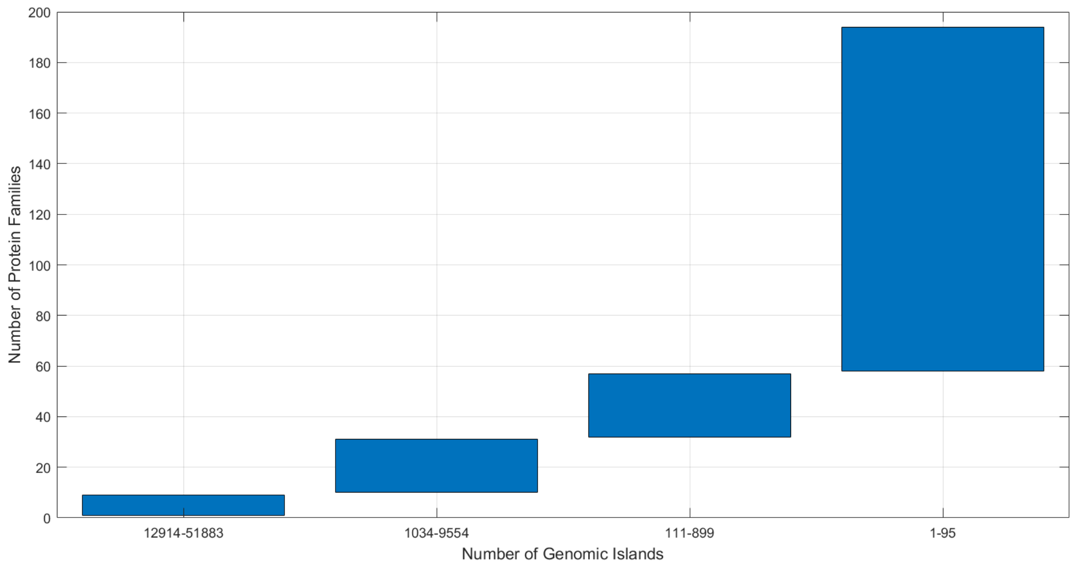

Figure S(1) –Bars represent the range of protein families among the categories that are represented by ranges of GIs

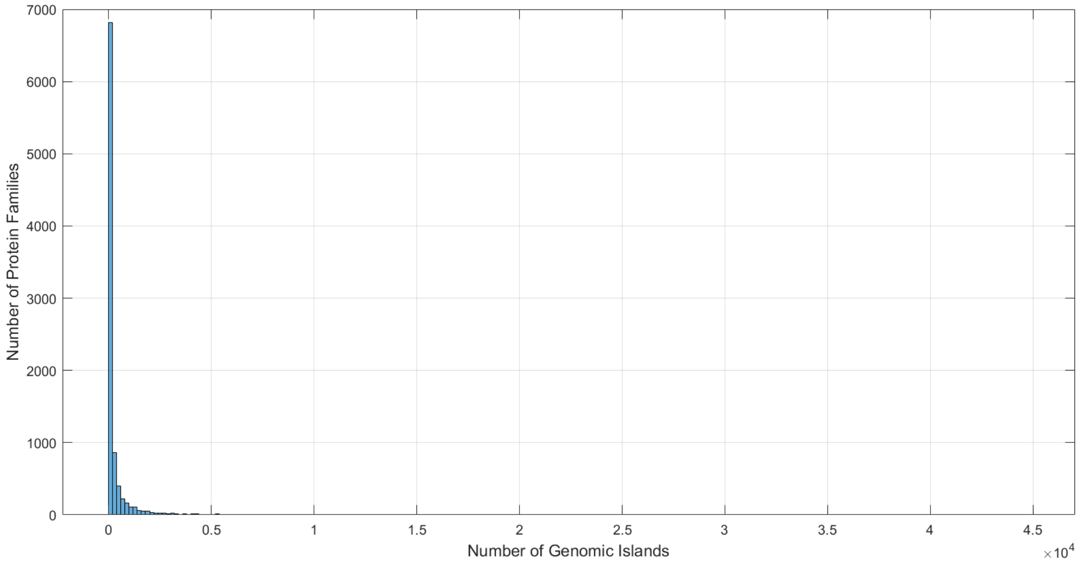

Figure S(2) –Histogram representing the distribution of protein families in genomic islands. Each bar represents the number of protein families in the genomic islands. In the beginning, there is a peak representing the number of 6816 protein families present in a number of genomic islands ranging from one to 200. In general, the chart shows that most protein families are present in a number of genomic islands, less than five thousand.

(in order).

The data set has a large number of protein families that are diverse in function. Figure S(2)

shows a histogram that represents the occurrence of protein families in GIs. In Figure S(2), most of the protein families in the data set exist in 2500 GIs or less. The protein families that are interesting are the ones that exist in most GIs.

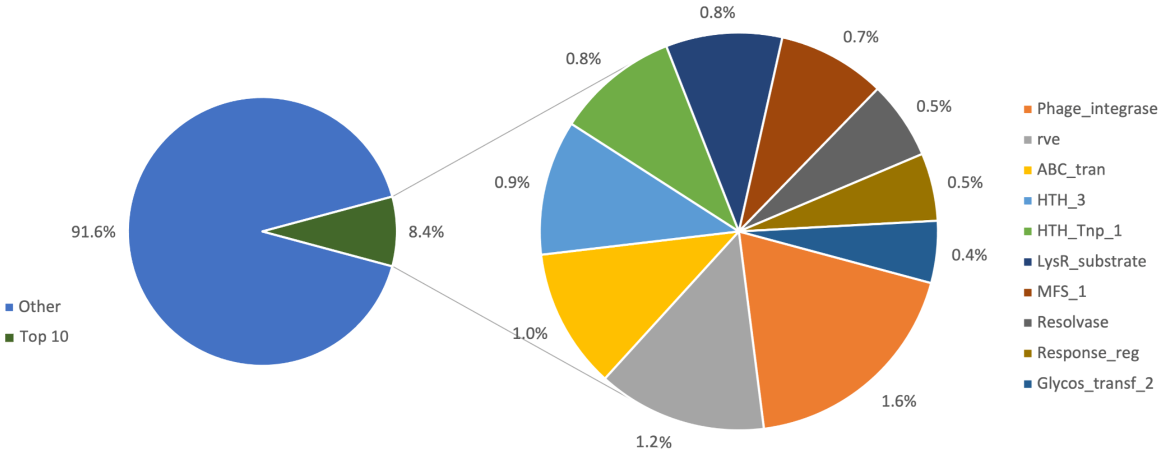

Figure S(3) –The top ten protein families exist in the GIs. Each percentage represents the proportion of occurrences of the protein family in the genomic islands.

Table S(1) –Information about the top ten protein families in the GIs

Protein Family	GIs	Pfam Clan	Gene Ontology (Molecular Function)
Phage_integrase	44605	DNA breaking-rejoining enzyme	DNA_binding
rve	32624	Ribonuclease H-like	-
ABC_tran	26987	P-loop containing nucleoside triphosphate hydrolase	ATP_binding
HTH_3	26010	Helix-turn-helix	-
HTH_Tnp_1	23636	Helix-turn-helix	DNA_binding, Transposase_activity
LysR_substrate	22401	Periplasmic binding protein	-
MFS_1	20781	Major Facilitator	Transmembrane_transporter_activity
Resolvase	15074	-	DNA_binding, DNA_strand_exchange_activity
Response_reg	13068	CheY-like	-
Glycos_transf_2	11942	Glycosyl transferase	-

Figure S(3) shows the top ten frequent protein families in the data set. More information about the top ten protein families can be found in Table S(1). The table shows the name of the protein family, the number of the GIs that have this protein family in their structure, the pfam clan (i.e. superfamily) of the protein family, and the gene ontology about the protein family.

According to the table, there are ten protein families with numerous functions, starting with the phage_integres protein family that exists in 44605 GIs. Phage integrases are enzymes whose main function is to catalyze the site-specific recombination between two sequences (i.e., DNA); the bacterial and phage attachment sites.

Below that, we have the rve protein family, short for the retroviral integrase. The retroviral integrase enzyme plays a significant role in a crucial phase in the replication cycle of viruses. Under high-salt conditions, retroviral intasomes have a great deal of resistance to challenges [28].

Under the rve protein family, there is the ABC_tran protein family. The ATP-binding cassette (ABC) transporters are known for being a large-sized superfamily of membrane proteins that contain diverse functions [23]. ABC transporters can be found in prokaryotes and play numerous roles for them. They represent efflux proteins and influx proteins. Efflux proteins are responsible for removing toxins from the cell, whereas influx proteins are responsible for transporting nutrients into the cell.To the present day, chemotherapy failure caused by ABC drug efflux is considered an ongoing research topic that frequently contributes additional evidence on multiple drug resistance, allowing scientists to tackle and overcome this matter. The efflux of drugs from the cell is the main mechanism of resistance performed by the membrane transporters that is found among all organisms. The membrane transporters are proteins that belong to the ABC transporter superfamily.Therefore, one of the fundamental causes of chemotherapy's unsuccess is the drug resistance that the prokaryotic ABC transporter family causes. Furthermore, bacterial cells also contain various families of ABC transporters which contribute to resistance to antibiotics [17].

The HTH_3 domain and HTH_Tnp_1 protein family both fall under the same superfamily called HTH, which consists of a large variety of principally DNA binding domains that include a helix-turn-helix motif. Many derivatives of the helix-turn-helix motif play a role in multiple antibiotic resistance, including the DNA-binding domain discovered in multiple antibiotic resistance regulators that form winged helix-turn-helix. Regarding HTH_Tnp_1, this protein family stands for helix-turn-helix transposase. The HTH structure is related to DNA binding, while transposase is needed for DNA transposition. Simply, HTH_Tnp_1 proteins bind to the nucleotide and assist in transposition of DNA. The HTH_Tnp_1 family contains many E. coli Insertion Elements (IS) along with various other bacterial transposases where some are members of the IS3 family, which can operate as a mobile promoter in E. coli [14].

The next protein family is the LysR_substrate protein family. The LysR_substrate family is a member of the clan Periplasmic Binding Proteins (PBPs). PBPs are nonenzymatic receptors that are used by bacteria to pick up small molecules and carry them into the cytoplasm. Most PBPs take part in transporting solute molecules to the cytoplasm by way of ABC transporters [42], where they aim for critical nutrients such as vitamins, amino acids, carbohydrates, and ions. Macheboeuf et al in [27] mentioned that PBPs play role in drug resistance.

Below that, the MFS_1 protein family can be seen, which belongs to the Major Facilitator Superfamily (MFS), it is one of the largest families of membrane transporters [29]. Since they take part in an essential role in several diseases by means of drug transportation, drug resistance, or aberrant action, members of the MFS family are central to the physiology of humans. The issue of resistance to antibiotics is often due to the action of MFS resistance genes.It has also been observed

that mutations in MFS transporters can cause diseases such as neurodegenerative disease [2], and glucose storage diseases [35]. Regarding MFS_1 in bacteria, the Arsenic Resistant (AR) bacteria species have evolved numerous efflux systems for AR. Many MFS proteins are submitted to UniProtKB, such as efflux pump antibiotic resistance (TARUN_5198) and MFS multidrug transporter (MFS_1).

Regarding the Resolvase protein family, in bacteria, resolvase proteins are commonly found on mobile DNA elements, such as plasmids and transposons. Antibiotic resistance genes are frequently associated with Tn3 family transposons [33].

Regarding the Response_reg protein family, the response regulators protein families form the central family of signaling proteins in prokaryotes. A protein from the response regulator's protein family assists the cell of the bacteria in responding to the environment changes. This is performed by enabling the bacteria to sense, respond, and adapt to numerous environments.There are some types of response regulators that are related to the resistance to antibiotics and protonophores. Concerning antibiotic resistance, QseB and BfmR response regulator proteins are responsible for degrees of antibiotic resistance in *Acinetobacter baumannii* and *Francisella novicida*, respectively [30]. Regarding protonophores resistance, the response regulator YcbB in Bacillus subtilis is one of the proteins whose disruption raises cell resistance to protonophores [38].

Finally, the Glycos_transf_2 protein family, glycosyl transferases (GTs), catalyse the formation of several kinds of glycoproteins with crucial roles in cell-to-cell recognition and communication [9]. One of their important roles is to catalyze the transfer of sugar onto aglycons, and this has a substantial association for the synthesis of natural products with high value.Glycans play significant roles in many biological processes in disease and health. There is a close relation between human and bacteria in the intestine, and this relation can be pathogenic or symbiotic. Bacterial GTs are considered as one of the virulence factors to humans. Therefore, understanding GTs has a significant role in vaccine production to protect against bacterial infections. Liposaccharide antibiotic moenomycin that inhibits bacterial glycosyltransferases is considered as a promising lead [47].

Regarding the Gene ontology, as shown in the table, not all Pfam families map directly to GO terms. As shown in the table, from the gene ontology view, the common molecular functions of the protein families are binding and transpose.

Table S(2) shows the top two protein families of the GIs in the data set, which means the most frequent two protein families that exist in the GIs together. Simply, as shown in Table S(2), proteins that usually belong to transpose protein families and binding or insertion protein families come together, such as in (HTH_Tnp_1, rve), (HTH_3, Phage_integrase), (ABC_tran, rve), (MFS_1, LysR_substrate). Furthermore, another case of the two protein families with the transpose and insertion or binding functionalities is the BPD_transp_1 and ABC_tran. BPD_transp_1 is a

Table S(2) –The top two protein families exist together in the GIs

Protein Families	The Number of GIs
HTH_Tnp_1, rve	11521
HTH_3, Phage_integrase	9730
BPD_transp_1, ABC_tran	7100
ABC_tran, rve	5882
HATPase_c, Response_reg	5512
IstB_IS21, rve	5218
MFS_1, LysR_substrate	5209
HTH_17, Phage_integrase	4744
Glycos_transf_1, Glycos_transf_2	4699
rve, Phage_integrase	4666

family that is a member of the clan BPD_transp_1, which is a clan containing families that are included in the transport of molecules across membranes. Regarding IstB_IS21, rve protein families, proteins belonging to the protein family IstB_IS21 contain an ATP/GTP binding P-loop motif. This motif is found to be linked to the IS21 family insertion sequences [41]. In general, The protein function is unknown, but there is the possibility that it can perform a transposase function [45]. There are some cases when we have two protein families with almost the same molecular function such as binding or insertion in the two protein families (rve, Phage_integrase), and (HTH_17, Phage_integrase).

Furthermore, in the (HATPase_c, Response_reg) case, the two protein families mainly function in sensing any changes in the environment and responding to these changes. HATPase_c belongs to the Histidine kinases clan and members of this superfamily are essential components of regulatory systems that allow bacteria to react to changes in their environment. Finally, Glycos_transf_1, Glycos_transf_2 protein families, overall, are from a large family of enzymes called Glycosyl transferases, as mentioned previously, they catalyze the transfer of sugars to a numerous of accept or molecules, which are important in all domains of life.

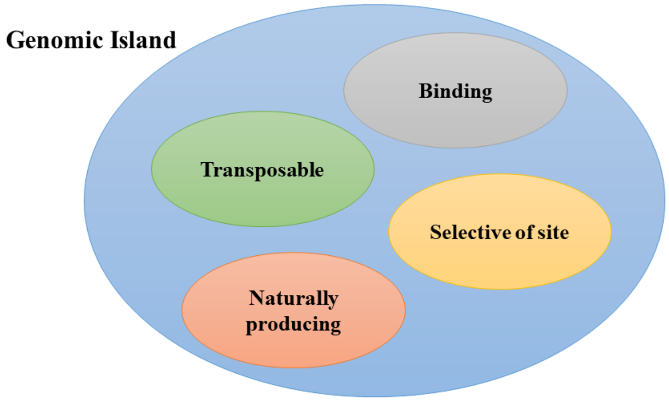

Figure S(4) –Main Functional Components in the GIs

To summarise, from all of the aforementioned information about the protein families that exist

in the GIs in the data set, it is highly possible that the main components of the GI are Transposable, Selective of site, Binding, and Naturally producing, as shown in Figure S(4). This makes sense as these are the main components that need to transfer a subsequence from one genome to another. The transpose function that is found in proteins belonging to protein families transposes facilities such as the MFS_1 protein family. After, there is the selection of site. The function of the proteins in these protein families is to specify the location of the subsequence in the host genome. An example of such a protein family is the rve. Regarding binding, which assists in binding the subsequence in the host genome, proteins with this function belong to protein families such as phage_integres protein family. Finally, there is the naturally producing, which could be possibly required for the success of the whole transfer process. An example of a protein family is Glycos_transf_2. It should be noted that the most important components are transposing and binding.

C Sets of Protein Families

Table S(3) –Top two protein families sets of each size

**Support**	**Size**	**Sets of Protein Families**	**Species**	**Genuses**
0.031277115	2	HTH_Tnp_1, rve	1102	438
0.02641525	2	Phage_integrase, HTH_3	1598	548
0.012076719	3	PapD_N, Fimbrial, Usher	206	42
0.01195907	3	Phage_tail_L, Lambda_tail_I, Phage_min_tail	190	60
0.008897242	4	Terminase_1, HNH, Phage_portal, Phage_capsid	465	178
0.008344289	4	Terminase_1, Phage_H_T_join, Phage_portal, Phage_capsid	471	186
0.007664864	5	Phage_tail_U, Phage_tail_T, Minor_tail_Z, Phage_TTP_12, Phage_TAC_2	48	20
0.00701485	5	Phage_H_T_join, HNH, Terminase_1, Phage_portal, Phage_capsid	371	148
0.006526605	6	Phage_tail_U, Phage_min_tail, Phage_tail_T, Minor_tail_Z, Phage_TTP_12, Phage_TAC_2	42	16
0.006438368	6	TMP_2, Phage_tail_U, Phage_tail_T, Minor_tail_Z, Phage_TTP_12, Phage_TAC_2	45	18
0.006097184	7	Phage_TTP_12, Phage_tail_U, Phage_tail_T, Minor_tail_Z, Phage_min_tail, Phage_tail_L, Phage_TAC_2	41	16
0.006100126	7	Phage_TTP_12, TMP_2, Phage_min_tail, Phage_tail_U, Minor_tail_Z, Phage_tail_T, Phage_TAC_2	42	16

Table S(3), shows the top two sets of each size and the support value for each pattern along with the number of species and genuses that have this set. For example, the first row represents a set of two protein families that exists in 3% GIs out of 368,339 GIs, along with 1102 species and 438 genuses. In general, Table S(3) shows numerous protein families in the patterns that are from different molecular functions. However, this table shows the protein families that most, if not all, of them have a very significant role in the horizontal gene transfer process. Starting with the phage tail protein families, the tail protein of phages is significant for the interaction between host

bacteria and phages. Phage tail proteins are responsible for host cell recognition and delivery of the viral genome to the host cytoplasm. For example, there are a number of minor tail proteins that have enzymatic activity. This type of activity assists the phage in recognizing the correct host. Furthermore, pass through the cell wall or surface to inject the DNA. The other important protein family is the Phage_portal family. The proteins of this protein family form a portal (i.e., hole or channel) that enables DNA passage during packaging and ejection. During the bacteria infection process, the protein family Phage_capsid plays a significant role in the success of this process since Phage_capsid proteins protect the viral genome during entry and exit from the host cells. Regarding Phage_integrase and rve proteins, they are responsible for the integration of a DNA copy of the viral genome into the host genome. Terminase_1 or Phage Terminase, the majority of the members of this family are phage proteins. In general, Terminase protein is a key component of the DNA packaging machine found in phages. Finally, the His-Asn-His (HNH) protein family is a common protein family that is mainly associated with endonuclease activity. It is a protein family that is composed of small nucleic acid-binding proteins. Endonucleases are enzymes that cleave the phosphodiester bond within a polynucleotide chain.

Table S(4) –The top ten protein families in the Sets

**Protein Family**	**The number of Sets exists in**
Phage_min_tail	152
Phage_TAC_2	137
Phage_tail_T	134
Phage_TTP_12	128
Phage_tail_L	123
Phage_tail_U	121
TMP_2	100
Minor_tail_Z	100
Lambda_tail_I	80
Phage_capsid	52

It was observed that the most frequent protein families in all sets are phage protein families, as shown in Table S(4). This could give an indication that the origin of the GIs could be from the phages.

D The "Phage_integrase, HTH_Tnp_1, rve" Pattern

The pattern (Phage_integrase, HTH_Tnp_1, rve) exists in species that belong to eleven different phyla, and that leads to them being dissimilar from one another, with each one having its own unique characteristics and living in different environments. Most of the species that have this pattern belong to the Proteobacteria, Actinobacteria, and Firmicutes phyla as shown in the bubble

Chart figure in the paper. Firstly, there is the *Candidatus Solibacter usitatus* which belongs to the Acidobacteria phylum. In the data set the *Candidatus Solibacter usitatus* strain that has this pattern is Ellin6076. *Candidatus Solibacter usitatus Ellin6076*, showed an abundance of genes that are affiliated with mobile genetic elements [12]. Furthermore, after comparative genome analyses, it was revealed that the Ellin6076 large genome came into being by HGT through the ancient phages or/and other processes [13]. The next phylum is Spirochaetes, where from this phylum, the species *Leptospira mayottensis* has this pattern in its genome. The *Leptospira mayottensis* species is a pathogenic species that comes from the genus Leptospira and is isolated from humans [8]. It was discovered in 2014 and is linked to various diseases [16]. Another species that has this pattern is *Kiritimatiella glycovorans*, which is from the Kiritimatiellaeota phylum. *Kiritimatiella glycovorans* is a species discovered in 2016. In general, the Kiritimatiellaceae family is composed of mainly bacteria which is found in environments that are hypersaline and anoxic. The species of Kiritimatiellaceae have a gram-negative cell wall that contains peptidoglycan (PNG).Gram-negative bacteria are the most prevalent primary pathogens. They possess multiple cell surface glycans that have demonstrated their importance in the process of the biosynthesis and regulation of the cell wall of pathogenic Gram-negative bacteria.The mechanical strength and shape of bacterial cell is owed to the peptidoglycan, which and can also play a role in the pathogenesis [6]. As a greatly conserved and vital constituent of almost every bacterial cell, PG is identified as a pathogen-associated molecular pattern by the eukaryotic immune system and is a potent activator of innate immunity [40]. Next, there is the species *Pleurocapsa* sp. PCC 7327 that belongs to the Cyanobacteria phyla. In general, the Cyanobacteria are found to be amongst the most diverse and widely dispersed phyla of bacteria.Moreover, they are essential contributors to global nitrogen fixation [46]. Found in a variety of marine environments, the genus Pleurocapsa is a nitrogen-fixing, spore-forming cyanobacterium, which can grow in either freshwater or saline environments. The other phylum that has species with this pattern is Nitrospirae, whose official name is *Nitrospira moscoviensis*. *Nitrospira moscoviensis* was discovered in 1995 and is a non-motile, non-marine, gram-negative, nitrite-oxidizing bacterium that has a curved rod shape [19]. The cytoplasm of *Nitrospira moscoviensis* contains polyhydroxybutyrate (granules), which are crucial storage compounds for carbon and energy in a number of prokaryotes, allowing for the survival of the cells when there is an absence of appropriate carbon sources [19]. The *Salinivirga cyanobacteriivorans* species that belongs to the Bacteroidetes phylum is another species that has this pattern. The species named *Salinivirga cyanobacteriivorans* was initially described by Ben Hania et al. 2017 [22]. It is a species of bacteria which preys on cyanobacteria. In general, the bacteria species of the Cyanobacteria phylum are gram-negative that obtain their energy by means of photosynthesis. Cyanobacteria also known as Cyanophyta, Deferribacteres phylum has a species named Denitrovibrio acetiphilus that has this pattern in its genome sequence. The species *Denitrovibrio acetiphilus* belong to the bacterial family Deferribacteraceae and bacterial genus Denitrovibrio [25].

Denitrovibrio acetiphilus has a curved rod-shaped structure and is a gram-negative, mesophilic, marine, anaerobic bacterium that respires by means of nitrate reduction. Its capacity to reduce nitrate is of economic significance, as it eradicates the requirement for expensive biocides that are presently used in the treatment of oil reserves [32]. Next, there is the *Deinococcus psychrotolerans* species that belongs to the Deinococcus-Thermus phylum. In 2019, *Deinococcus psychrotolerans* was identified [43]. It is a coccus-shaped, non-motile, gram-negative, strictly aerobic bacterium. The species of the genus Deinococcus are characterized by their resistance to ionizing radiation.

After that, there is the species *Achromobacter denitrificans* from the Proteobacteria phylum. Formerly, the *Achromobacter denitrificans* species was known as *Alcaligenes denitrificans*. It was recently reclassified under the Achromobacter genus [11]. The *Achromobacter denitrificans* species are gram-negative, motile, strictly aerobic, and ubiquitous bacterium. The strains of *Alcaligenes denitrificans* exist in soil; however, they can sometimes exist in human clinical samples since they can cause human infections [15]. Later, in the Actinobacteria phylum it has been discovered that the *Gordonia bronchialis* species have this pattern. Gordonia species are Gram-positive and aerobic actinomycetes that have recently been recognized for causing human disease. The species that belong to the Gordonia genus are gram-positive, catalase-positive, nonmotile, and aerobic [5]. There are numerous Gordonia species that have been isolated from soil [39]. Furthermore, the bacterium species *Gordonia bronchialis* was discovered in a number of patients with sternal wounds, and skin [44].Numerous laboratories for clinical microbiology could misidentify Gordonia species for Rhodococcus and Nocardia species. This is due to the fact that the species that belong to the Gordonia genus are closely related to the species that belong to the Rhodococcus genus and Nocardia genus. In the data set there species from Nocardia or Rhodococcus that have this pattern.

Finally, there is the species *Halanaerobium hydrogeninformans* from the phylum Firmicutes. *Halanaerobium hydrogeninformans* is an obligatory anaerobic, gram positive, non motile, elongated rod-shaped bacterium, that is able to tolerate exceptionally high salinity and high alkalinity conditions within its environment [10]. Most commonly found in halo alkaline lakes, *Halanaerobium hydrogenoformans* is an alkaliphilic bacterium that can carry out biohydrogen production. Microorganisms are currently being explored as a means of chemical and biofuel production, such as future biohydrogen generation at industrial scales. Owing to the increase in price of fossil fuels and diminishing of reserves, biofuel production is viewed as a viable contribution to present and future energy demands. *Halanaerobium hydrogeninformans* are able to use a number of pure sugars for hydrogen production, and, consequently, this bacterium is potentially capable of raising the level of efficiency and efficacy of biohydrogen production that comes from renewable biomass resources [10].

E Compound connection

The bacteria in this compound connection are *Citrobacter amalonaticus*, *Enterobacter hormaechei*, *Salmonella enterica* and all have their own special characteristics. The *Citrobacter amalonaticus* belongs to the Citrobacter genus, which is a genus of aerobic, gram-negative bacterium that is part of the Enterobacteriaceae family. Citrobacter species have been found in sewage, water bodies, the human gut, as well as in animal intestines [31]. The existence of Citrobacter species in sewage water is seen as a significant public health threat, especially in areas heavily populated with humans, due to the capacity of these bacteria to spread efficiently and quickly once an infection takes place. Infection from Citrobacter species is associated with urinary tract infections, gastroenteritis, and neonatal meningitis [26]. *Citrobacter amalonaticus*, or *Levinea amalonatica*, was initially described by Young et al. in 1971. The *Citrobacter amalonaticus* Y19 strain was reported to have the capability of producing hydrogen from oxidizing toxic carbon monoxide [1]. Regarding *Enterobacter hormaechei*, it is a species of gram-negative, oxidase-negative bacteria which is extensively found in the majority of temperate soils and waters [34]. Overall, Enterobacter species are widely scattered across many areas such as soil, water, vertebrate and invertebrate hosts, and in the feces of animals and humans. *Enterobacter hormaechei* is also an opportunistic infectious pathogen, as it can be present within the intestinal tract of humans causing diseases in immunocompromised hospital patients [18].Moreover, *Enterobacter hormaechei* infections have been discovered in a variety of mammals, such as pets with respiratory disease complex [24].Finally, the *Salmonella enterica*, in general, Salmonella is a gram-negative bacteria with a rod-shaped appearance and facultative anaerobes that belong to the Enterobacteriaceae family. Salmonella species have the ability to multiply and grow under numerous environmental conditions outside the living hosts. Salmonella genus, which is closely related to the genus Escherichia, is known as the most prevalent foodborne pathogen that is often detached from food-producing animals, causing zoonotic infections in humans and various animal species including birds. Thus, infections from Salmonella are a major concern to public health, the health of animals, as well as the global food industry [3]. The intestinal tract of humans and farm animals is Salmonella serovars' main niche. However, it can also be found in the intestinal tract of reptiles, wild birds and less commonly insects [4]. Salmonella is divided into two different groups, *Salmonella bongori* and *Salmonella enterica. Salmonella enterica* species is a food-borne pathogen known for causing mild to serious human diseases, such as mild gastroenteritis and severe systemic infections. One of the major disease burdens worldwide is the human infections caused by *Salmonella enterica* through contaminated water or food. *Salmonella enterica* is like the other salmonella species that cause foodborne illness globally and is accountable for significant public health as well as economic damages. When testing for *Salmonella enterica* in food, there are usually issues caused by the existence of background microflora that could present themselves as Salmonella which is false-positive [20]. It is often very challenging to tell the difference between false-positive isolates that belong to the Citrobacter genus and Salmonella genus owing

to the similarities in their cell surface antigens, genetics, as well as other phenotypes [36]. The core genome analysis shows that the Citrobacter and *Salmonella enterica* populations investigated appear to share a common evolutionary history. As with Salmonella, Citrobacter is often isolated from water, soil, and animals' digestive tract [7]. In general, an ambiguous connection between *Escherichia coli*, *Citrobacter*, and *Salmonella* is apparent, which presents a practical issue in the field of medical diagnosis and food safety testing, since the identification of accurate species is crucial to confirm the existence of pathogens [36].

F Analysis of the Phages in the connections

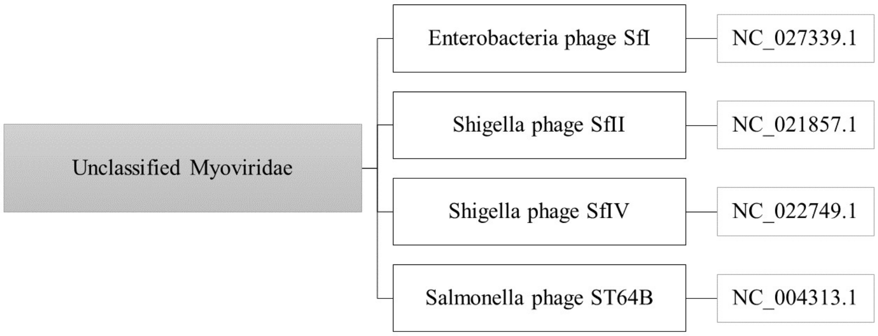

Figure S(5) –Case 1 phages phylogeny

In this section, the phages in the connections were analyzed. The *Connections* table shows that there is a group of phages in a connection with the same bacteria species. For example, there is a connection between Phage MF695815.1 and bacteria NZ_LS992183.1, NZ_CP020820.1. Furthermore, the same bacteria exists in another connection with phage MK416014.1. In the *Connections* table there are three cases where a group of phages is in HGT connection with the same bacteria species. Therefore, phylogenetic trees and BLAST analysis were performed to analyze the phages and understand the relation between these phages. Starting with the first case, blue rows, Figure S(5) shows the phylogeny connection between the phages in the first case in the table. The figure shows the four phages are from different species. All the species belong to the same taxonomy level named unclassified Myoviridae. Unclassified Myoviridae is a level with no rank under the Myoviridae family.

Connections (Phages BLAST Analysis) table in the Supplemental document shows the BLAST analysis for all the phages in the *Connections* table. The BLAST analysis shows that the E value is zero between all the phages regarding the first case. The second case (i.e., orange) in the *Connections* table includes two partial genomes that belong to the same species; *uncultured Caudovirales phage*. In the *Connections (Phages BLAST Analysis)* table, the idetity value is 99.982% and the E value equated to zero. Moreover, the query coverage is around 100%. It is worth mention-

ing that the difference between the length of the two phage genomes (MF417956.1=16570 bp, MF417957.1=16537 bp) is very small. Finally, the last case include species from the same taxonomy level named unclassified Siphoviridae. In the BLAST analysis, as shown in the *Connections (Phages BLAST Analysis)* table, the identity values above 98% and the E values equated to zero.

Overall, it is obvious from the coverage values in each case that there is a common subsequence between the phages in each case. This sequence could be the GI in each connection in the phage and bacteria. More information about the shared subsequence (i.e. GI) is presented in the *The Existence of a Shared Prokaryotic GI in the connection Species* Section in the main text.

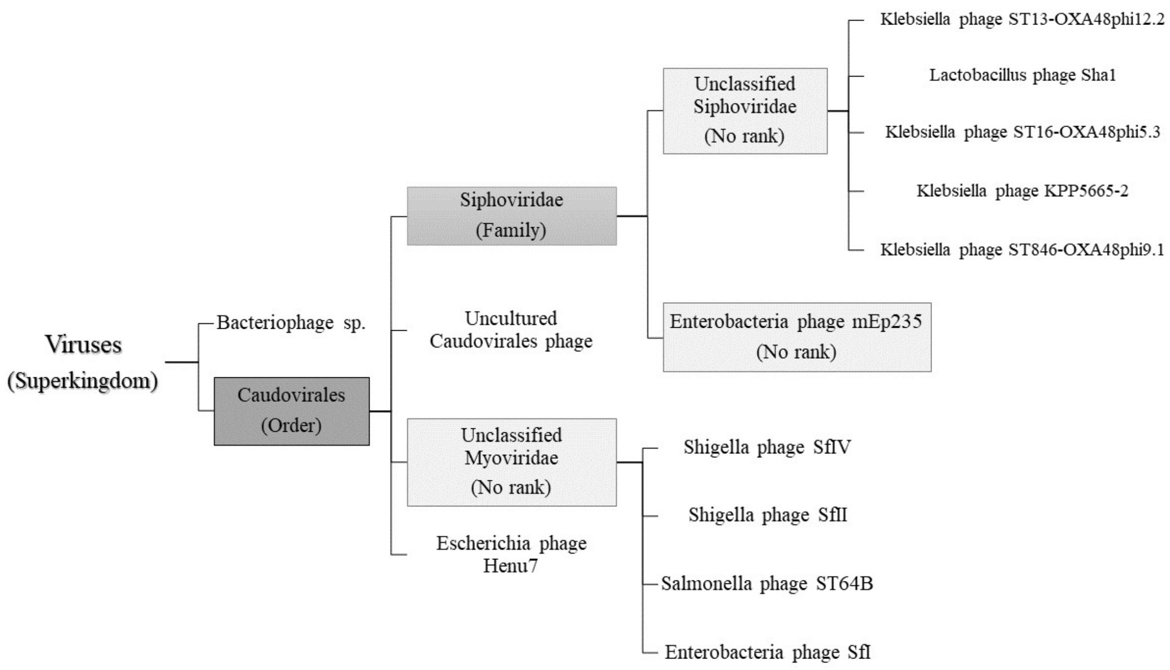

Figure S(6) –Connections phages phylogeny

The phylogeny of the phages in the connections is presented in Figure S(6). The phylogeny shows that most of the phages belong to the *Caudovirales* order except *Bacteriophage sp.*. In general, it is clear that there is a connection between phages in each case, and this connection deserves further investigation in the future.

G Supplemental Algorithms

See the main document for the purpose of these algorithms and for the references to the pseudocode by algorithm number.

Algorithm 1: Patterns

```
Input: GIs, GIs_Proteins
Output: Patterns Files
Apriori_Sets ← Apriori(GIs Proteins)
Patterns=[]
for S in Apriori_Sets do
    Set_Patterns=itertools.permutations(S)
    for SP in list(Set_Patterns) do
        Patterns.append(SP)
    end
end
Interesting_Patterns=[]
for P in Patterns do
    if 3 ≤ length(P) ≤ 5 then
        Pattern_GIs=[]
        for GI in GIs do
            if P in GI then
                Pattern_GIs.append(GI)
            end
        end
        (Superkingdom,Phylum,Class,Order,Family,Genus,Species) ← Taxonomy(Pattern_GIs)
        if len(Pattern_GIs) > 10 then
            if Most_Frq_Order = 7 then
                Interesting_Patterns.append(P,Superkingdom,Phylum,Class,Order,Family,Genus,Species,
                  len(Pattern_GIs))
            end
        end
    end
end
return Interesting_Patterns
```

Algorithm 2: Presence of Phages in the Patterns

```
Input: Patterns Files
Output: Patterns_Phages Proteins Files
for P in Patterns do
    Pattern_GIs ← Retrieve_GIs(P)                      ▷ Retrieve the GIs that have the P pattern
    for GI in Pattern_GIs do
        GI_Proteins ← Pattern_GI_Proteins(GI,P)        ▷ Retrieve the proteins of the GI
        InputFile_GI_Proteins=open(Input_File.txt,w)
        InputFile_GI_Proteins.write(GI_Proteins)
        OutputFile_GI_Viral_Proteins=open(P+'_'+GI+'.txt',w)
        blastp -db Viral_DataBase -query InputFile_GI_Proteins -out OutputFile_GI_Viral_Proteins
          -evalue 10e-10
    end
end
```

Algorithm 3: Patterns — Phage Information

```
Input: Patterns_PhagesProteins Files
Output: Patterns_PhagesProteinsInfo Files
for P in Patterns do
    PatternFile=open(P+'.txt')
    Pattern_GIs ← Retrieve_GIs(P)
    for GI in Pattern_GIs do
        File=read(P+'_'+GI+'.txt')
        index = −1
        BP =''
        for Line in File do
            (BacteriaProtein, PhageProteinAccessoin, Evalue) = Get_Line_Data(Line)
            (OrganismName , OrganismAccession)=EFetch(PhageProteinAccessoin)
            if index==-1 or BacteriaProtein != BP then
                index=index+1
                Organism_Name.append([])
                Organism_Acc.append([])
                Protein_Acc.append([])
                Protein_Evalue.append([])
            end
            Organism_Name[index].append(OrganismName)
            Organism_Acc[index].append(OrganismAccession)
            Protein_Acc[index].append(PhageAccessoin)
            Protein_Evalue[index].append(Evalue)
            BP=BacteriaProteiny
        end
        if index ==length(P) then
            GI_Phage_Intersection=Intersection(Organism_Acc)
            for Phage in GI_Phage_Intersection do
                PatternFile.write(GI, Phage, Max(E value))
            end
        end
    end
end
```

Algorithm 4: Extracting HGT Connections

```
Input: Patterns_PhagesProteinsInfo Files
Output: Phage_Bacteria_Connections File
for P in Patterns do
    PatternFile=read(P+'.txt')
    PatternPhageInfo=write('PhagesInGIs'+P+'.txt')
    Phages=[], Seen=[], P_Phages=[], P_GIs=[], P_Evalue=[]
    for Line in PatternFile do
        (Phage, GI, Evalue) ← Get_Data(Line)
        P_Phages.append(Phage),   P_GIs.append(GI),   P_Evalue.append(Evalue)
        if Phage not in Seen then
            Phages.append(Phage),   Seen.append(Phage)
        end
    end
    for Phage in Phages do
        P_Index=0, Bacteria_GIs=[], Evalues=[]
        for PP in P_Phages do
            P_Index+=1
            if Phage == PP then
                Bacteria_GIs.append(P_GIs[P_Index])
                Evalues.append(P_Evalue[P_Index])
            end
        end
        PatternPhageInfo.write(P, Phage, Bacteria_GIs, Evalues)
    end
end
```

Algorithm 5: Filtering HGT Connections — Taxonomy

```
Input: Phage_Bacteria_connections File
Output: Phage_Bacteria_connections_TaxFilter File
for P in Interesting_Patterns do
    PatternFile=read('PhagesInGIs'+P+'.txt')
    GIsLineageFile=write('GIsLineage'+P+'.txt')
    GIsEvaluesFile=write('GIsLineageEvalues'+P+'.txt')
    for Line in PatternFile do
        Bacteria_List ← Get_Bacteria(Line)
        Lineage=[]
        for B in Bacteria_List do
            Lineage.append(Get_Lineage(B))
        end
        Common_Lineage=Intersection(Lineage)
        if Length (Common_Lineage) <5 then
            GIsLineageFile.write(Line, Common_Lineage)
            Bacteria_GIs=[],    Bacteria_Evalues=[]
            for B in Bacteria_List do
                if Evalue <10e-100 then
                    Bacteria.append(B)
                end
            end
            Phage ← GetPhage(Line)
            GIsEvaluesFile.write(Phage, Bacteria_GIs, Bacteria_Evalues)
        end
    end
end
```

Algorithm 6: Connections

```
Input: Phage_Bacteria_connections_TaxFilter File
Output: Novel_HGT_connections File
PatternFile=read('GIsLineageEvalues'+P+'.txt')
Phages_vs_Bacteria=write(PhagesVSBacteria'+P+'.txt')
Bacteria_vs_Bacteria=write(BacteriaVSBacteria'+P+'.txt')
HGT=write(HGT.txt)
for Line in PatternFile do
    (Phage, GIs, Bacteria_Name) ← Get_Data(Line)
    (Phage_Proteins) ← Get_Proteins(Phage)
    (Bacteria_Proteins) ← Get_Proteins(GIs)
    Flage1=0, Flage2=0
    PhageVSBacteria=BLAST(Phage_Proteins , Bacteria_Proteins)
    for FB in PhageVSBacteria do
        if (FB.ScientificName == Bacteria_Name) and (FB.Identity ≥ 85) and
          (TextSimilarity(FB.ScientificName,Bacteria_Name) <0.4) then
            Phages_vs_Bacteria.write(Phage, FB.GI)
            Flage1=1
        end
    end
    BacteriaVSBacteria=BLAST_BothDirections(Bacteria_Proteins , Bacteria_Proteins)
    for FB in PhageVSBacteria do
        if (FB.ScientificName == Bacteria_Name) and (FB.Identity ≥ 85) and
          (TextSimilarity(FB.ScientificName,Bacteria_Name) <0.4) then
            Bacteria_vs_Bacteria.write(FB.GI, FB.GI)
            Flage2=1
        end
    end
    if Flage1==1 and Flage2==1 and ((Phage, GIs) ∩ (GIs, GIs)) ≠ ∅ then
        HGT.write((Phage,GIs),(GIs,GIs))
    end
end
```

Algorithm 7: Connections — Extract Species Subsequences

```
Input: Novel_HGT_connections File
Output: Blast Results, Clustal
for connection in connectionsFile do
    for i in connectionsBacteria do
        Coordinates ← BLAST(Phage,Bacteria)
    end
    //Get Phage Coordinates
    if Phage_coordinates_Equal: then
        (Start, End) ← Get_any_phage_coordinates()
    end
    else if Phage_Starts_Equal then
        End ← Get_Phage_coordinates_End()
    end
    else if Phage_Ends_Equal then
        Start ← Get_Phage_coordinates_Start()
    end
    else
        //Phage_coordinates_not_Equal
        (Start, End) ← Compute_phage_coordinates()
    end
    Phage subsequence ← Extract_Phage_subsequence (Start, End)
    PhageFile ← write(Phage subsequence)
    ClustalFile ← write(Phage subsequence)
    //Get Bacteria Coordinates
    if length(Bacteria)==1 then
        //Onebacteria
        Clustal ← write(Bacteria Subsequence)
    end
    else
        //More than one bacteria
        for i in Bacteria do
            Difference = abs((PhageS − PhageE + 1) − (abs(Bacterium_E − Bacterium_S) + 1))
            X = Difference/2;  A = int(X);  Y = X − A
            if Bacteria_Phage_Length_Difference == 0 then
                Clustal ← write(Bacteria Subsequence)
            end
            else if Bacteria_Phage_Length_Difference > 1200 then
                New_Coordinates ← BLAST(phage, bacteria(i))
                Clustal ← write(Bacteria Subsequence (New_Coordinates))
            end
            else if Bacteria_Phage_Length_Difference > 0 then
                Clustal ← write(Bacteria_Subsequence_Extend)
            end
            else
                Clustal ← write(Bacteria_Subsequence_Trim)
            end
        end
    end
end
```

H Supplemental Tables

Table S(5) –Top species in the data set

Species	Genomic Islands
Escherichia coli	51152
Salmonella enterica	29225
Klebsiella pneumoniae	17238
Bordetella pertussis	16382
Pseudomonas aeruginosa	8614

Table S(6) –Patterns

Index	Pattern Size	Pattern- Protein Family Name	Pattern- Protein Family Accession
1	3	Phage_capsid HK97-gp10_like HNH	PF05065.14 PF04883.13 PF01844.24
2	3	Terminase_1 HK97-gp10_like HNH	PF03354.16 PF04883.13 PF01844.24
3	3	Terminase_1 Phage_capsid HK97-gp10_like	PF03354.16 PF05065.14 PF04883.13
4	3	Phage_H_T_join Terminase_1 HNH	PF05521.12 PF03354.16 PF01844.24
5	3	HNH Phage_portal Phage_capsid	PF01844.24 PF04860.13 PF05065.14
6	3	Phage_capsid HNH Phage_portal	PF05065.14 PF01844.24 PF04860.13
7	3	Phage_capsid Phage_portal HNH	PF05065.14 PF04860.13 PF01844.24
8	3	HNH Terminase_1 Phage_capsid	PF01844.24 PF03354.16 PF05065.14
9	3	Terminase_1 Phage_capsid HNH	PF03354.16 PF05065.14 PF01844.24
10	3	Phage_capsid HNH Terminase_1	PF05065.14 PF01844.24 PF03354.16
11	3	Terminase_4 Phage_capsid HNH	PF05119.13 PF05065.14 PF01844.24
12	3	Phage_connect_1 Phage_portal HNH	PF05135.14 PF04860.13 PF01844.24
13	3	HNH Terminase_1 Phage_portal	PF01844.24 PF03354.16 PF04860.13
14	3	Terminase_4 Terminase_1 HNH	PF05119.13 PF03354.16 PF01844.24
15	3	HTH_Tnp_1 rve Phage_integrase	PF01527.21 PF00665.27 PF00589.23
16	3	Phage_integrase HTH_Tnp_1 rve	PF00589.23 PF01527.21 PF00665.27
17	3	Terminase_1 Phage_portal Phage_capsid	PF03354.16 PF04860.13 PF05065.14
18	3	Terminase_1 Phage_capsid Phage_portal	PF03354.16 PF05065.14 PF04860.13
19	3	Phage_capsid Phage_portal Terminase_1	PF05065.14 PF04860.13 PF03354.16
20	4	Terminase_1 Phage_capsid HK97-gp10_like HNH	PF03354.16 PF05065.14 PF04883.13 PF01844.24

Table S(7) –Connections. Each row represents a HGT connection. This means a phage and bacteria that have a pattern in common. The header shows the pattern index number, the pattern protein families, the phage name, the phage genome accession number, and the bacteria genome accession numbers. Rows colored with the same color mean that there are different phages have a connection with the same group of bacteria.

Index	Pattern	Phage Name	Phage Genome	Bacteria Genomes
44	Terminase_1 Phage_portal Phage_capsid	*Bacteriophage* sp.	MN855837.1	NZ_CP026116.1, NZ_AP017931.1
54	Terminase_1 Phage_portal Phage_capsid	*Enterobacteria* phage SfI	NC_027339.1	NZ_CP030787.1
58	Terminase_1 Phage_portal Phage_capsid	*Enterobacteria* phage mEp235	NC_019708.1	NC_009778.1
64	Terminase_1 Phage_portal Phage_capsid	*Escherichia* phage Henu7	LR881103.1	NZ_CP035214.1, NC_015968.1, NZ_CP020388.1
73	Terminase_1 Phage_portal Phage_capsid	*Klebsiella* phage ST13-OXA48phi12.2	MK422452.1	NZ_LT556084.1
92	Terminase_1 Phage_portal Phage_capsid	*Salmonella* phage ST64B	NC_004313.1	NZ_CP030787.1
94	Terminase_1 Phage_portal Phage_capsid	*Shigella* phage SfII	NC_021857.1	NZ_CP030787.1
96	Terminase_1 Phage_portal Phage_capsid	*Shigella* phage SfIV	NC_022749.1	NZ_CP030787.1
200	Terminase_1 Phage_portal Phage_capsid	*Uncultured Caudovirales* phage clone 7S_14	MF417956.1	NZ_CP035214.1, NC_015968.1, NZ_CP020388.1
201	Terminase_1 Phage_portal Phage_capsid	*Uncultured Caudovirales* phage clone 2AX_6	MF417957.1	NZ_CP035214.1, NC_015968.1, NZ_CP020388.1
383	Phage_capsid Phage_portal Terminase_1	*Klebsiella* phage ST13-OXA48phi12.2	MK422452.1	NZ_CP037894.1, NZ_CP032841.1
385	Phage_capsid Phage_portal Terminase_1	*Klebsiella* phage ST846-OXA48phi9.1	MK416021.1	NZ_CP020448.2, NZ_CP038469.1
571	Terminase_1 Phage_portal Phage_capsid	*Klebsiella* phage KPP5665-2	MF695815.1	NZ_LS992183.1, NZ_CP020820.1
573	Terminase_1 Phage_portal Phage_capsid	*Klebsiella* phage ST16-OXA48phi5.3	MK416014.1	NZ_LS992183.1, NZ_CP020820.1
1385	HNH Phage_portal Phage_capsid	*Lactobacillus* phage Sha1	NC_019489.1	NZ_CP021927.1

Table S(8) –Connections (Phages BLAST Analysis)

CI1	CI2	Phage1 Acc.	Phage2 Acc.	Identity	E Value	Ph1 Len	Ph2 Len	Overlap	Cov. 1	Cov. 2
54	58	NC_027339.1	NC_019708.1	94.737	2.43E-160	38389	37595	361	1%	1%
54	92	NC_027339.1	NC_004313.1	83.345	0	38389	40149	9649	25%	24%
54	94	NC_027339.1	NC_021857.1	98.164	0	38389	41475	12093	32%	29%
54	96	NC_027339.1	NC_022749.1	98.186	0	38389	39758	6008	16%	15%
58	73	NC_019708.1	MK422452.1	79.772	0	37595	34141	3421	9%	10%

Continued on next page

Table 8 – continued from previous page

CI1	CI2	Phage1 Acc.	Phage2 Acc.	Identity	E Value	Ph1 Len	Ph2 Len	Overlap	Cov. 1	Cov. 2
58	94	NC_019708.1	NC_021857.1	88.354	9.77E-135	37595	41475	395	1%	1%
58	96	NC_019708.1	NC_022749.1	87.342	4.39E-128	37595	39758	395	1%	1%
58	385	NC_019708.1	MK416021.1	75.261	0	37595	38370	1722	5%	4%
58	571	NC_019708.1	MF695815.1	75.134	0	37595	39241	1673	4%	4%
64	200	LR881103.1	MF417956.1	90.841	0	16548	16570	3188	19%	19%
64	201	LR881103.1	MF417957.1	90.841	0	16548	16537	3188	19%	19%
64	571	LR881103.1	MF695815.1	79.545	1.41E-159	16548	39241	792	5%	2%
73	571	MK422452.1	MF695815.1	92.448	4.87E-157	34141	39241	384	1%	1%
73	573	MK422452.1	MK416014.1	92.664	0	34141	29301	1445	4%	5%
92	94	NC_004313.1	NC_021857.1	83.591	0	40149	41475	9629	24%	23%
92	96	NC_004313.1	NC_022749.1	82.286	0	40149	39758	11031	27%	28%
94	96	NC_021857.1	NC_022749.1	97.284	0	41475	39758	6739	16%	17%
200	201	MF417956.1	MF417957.1	99.982	0	16570	16537	16537	99%	100%
385	571	MK416021.1	MF695815.1	92.308	1.46E-08	38370	39241	39	0%	0%
385	573	MK416021.1	MK416014.1	81.407	1.26E-82	38370	29301	398	1%	1%
571	573	MF695815.1	MK416014.1	98.891	0	39241	29301	8385	21%	29%

Table S(9) –Connections (Phages and Bacteria BLAST Analysis)

CI	Species Name	Species Accession	Species Name	Species Accession	Ident.
44	Bacteriophage_sp.	MN855837.1	Lactobacillus_curvatus	NZ_CP026116.1	90.268
44	Bacteriophage_sp.	MN855837.1	Lactobacillus_sakei	NZ_AP017931.1	93.356
44	Lactobacillus_curvatus	NZ_CP026116.1	Lactobacillus_sakei	NZ_AP017931.1	93.705
54	Enterobacteria_phage_SfI	NC_027339.1	Escherichia_albertii	NZ_CP030787.1	96.715
58	Enterobacteria_phage_mEp235	NC_019708.1	Cronobacter_sakazakii	NC_009778.1	80.314
64	Escherichia_phage_Henu7	LR881103.1	Klebsiella_michiganensis	NZ_CP035214.1	94.267
64	Escherichia_phage_Henu7	LR881103.1	Enterobacter_soli	NC_015968.1	90.602
64	Escherichia_phage_Henu7	LR881103.1	Pluralibacter_gergoviae	NZ_CP020388.1	87.451
64	Klebsiella_michiganensis	NZ_CP035214.1	Enterobacter_soli	NC_015968.1	90.365
64	Klebsiella_michiganensis	NZ_CP035214.1	Pluralibacter_gergoviae	NZ_CP020388.1	82.271
64	Enterobacter_soli	NC_015968.1	Pluralibacter_gergoviae	NZ_CP020388.1	94.589
73	Klebsiella_phage_ST13-OXA48phi12.2	MK422452.1	Citrobacter_amalonaticus	NZ_LT556084.1	80.409
92	Salmonella_phage_ST64B	NC_004313.1	Escherichia_albertii	NZ_CP030787.1	84.897
94	Shigella_phage_SfII	NC_021857.1	Escherichia_albertii	NZ_CP030787.1	96.734
96	Shigella_phage_SfIV	NC_022749.1	Escherichia_albertii	NZ_CP030787.1	96.989
200	uncultured_Caudovirales_phage	MF417956.1	Klebsiella_michiganensis	NZ_CP035214.1	88.169
200	uncultured_Caudovirales_phage	MF417956.1	Enterobacter_soli	NC_015968.1	94.426
200	uncultured_Caudovirales_phage	MF417956.1	Pluralibacter_gergoviae	NZ_CP020388.1	90.268
200	Klebsiella_michiganensis	NZ_CP035214.1	Enterobacter_soli	NC_015968.1	90.365
200	Klebsiella_michiganensis	NZ_CP035214.1	Pluralibacter_gergoviae	NZ_CP020388.1	82.271
200	Enterobacter_soli	NC_015968.1	Pluralibacter_gergoviae	NZ_CP020388.1	94.589

Continued on next page

Table 9 – continued from previous page

CI	Species Name	Species Accession	Species Name	Species Accession	Ident.
201	uncultured_Caudovirales_phage	MF417957.1	Klebsiella_michiganensis	NZ_CP035214.1	88.169
201	uncultured_Caudovirales_phage	MF417957.1	Enterobacter_soli	NC_015968.1	94.426
201	uncultured_Caudovirales_phage	MF417957.1	Pluralibacter_gergoviae	NZ_CP020388.1	90.268
201	Klebsiella_michiganensis	NZ_CP035214.1	Enterobacter_soli	NC_015968.1	90.365
201	Klebsiella_michiganensis	NZ_CP035214.1	Pluralibacter_gergoviae	NZ_CP020388.1	82.271
201	Enterobacter_soli	NC_015968.1	Pluralibacter_gergoviae	NZ_CP020388.1	94.589
383	Klebsiella_phage_ST13-OXA48phi12.2	MK422452.1	Salmonella_enterica	NZ_CP037894.1	80.085
383	Klebsiella_phage_ST13-OXA48phi12.2	MK422452.1	Enterobacter_hormaechei	NZ_CP032841.1	81.947
383	Salmonella_enterica	NZ_CP037894.1	Enterobacter_hormaechei	NZ_CP032841.1	90.646
385	Klebsiella_phage_ST846-OXA48phi9.1	MK416021.1	Citrobacter_braakii	NZ_CP020448.2	77.904
385	Klebsiella_phage_ST846-OXA48phi9.1	MK416021.1	Citrobacter_tructae	NZ_CP038469.1	77.961
385	Citrobacter_braakii	NZ_CP020448.2	Citrobacter_tructae	NZ_CP038469.1	91.507
571	Klebsiella_phage_KPP5665-2	MF695815.1	Citrobacter_freundii	NZ_LS992183.1	84.107
571	Klebsiella_phage_KPP5665-2	MF695815.1	Pantoea_vagans	NZ_CP020820.1	78.460
571	Citrobacter_freundii	NZ_LS992183.1	Pantoea_vagans	NZ_CP020820.1	91.307
573	Klebsiella_phage_ST16-OXA48phi5.3	MK416014.1	Citrobacter_freundii	NZ_LS992183.1	83.455
573	Klebsiella_phage_ST16-OXA48phi5.3	MK416014.1	Pantoea_vagans	NZ_CP020820.1	78.541
573	Citrobacter_freundii	NZ_LS992183.1	Pantoea_vagans	NZ_CP020820.1	91.307
1385	Lactobacillus_phage_Sha1	NC_019489.1	Pediococcus_pentosaceus	NZ_CP021927.1	88.159

Table S(10) –Connections (GIs Coordinates Information). The table includes the length of the two genomes; the query length (Q length) and subject length (S length), the overlap or alignment length (A Len), the start and the and of query alignment (Q Start, Q End), the start and the and of subject alignment (S Start, S End), the start and the end of the GI that is discovered in the bacteria genome (i.e. subject genome) as mentioned in the IslandViewer4 website.

CI	Q Species	S Species	Q length	S length	A Len	Q Start	Q End	S Start	S End	GI Start	GI End
44	MN855837.1	NZ_CP026116.1	10442	1898076	5004	1	5001	723582	718580	698993	744389
44	MN855837.1	NZ_AP017931.1	10442	1950487	4997	1	4997	477956	482951	469294	485205
44	NZ_CP026116.1	NZ_AP017931.1	1898076	1950487	15124	1340969	1356052	129474	114392	-	-
54	NC_027339.1	NZ_CP030787.1	38389	4659718	5875	2	5876	1010412	1016286	994657	1036029
58	NC_019708.1	NC_009778.1	37595	4368373	5801	1	5757	988865	994638	956100	1008032
64	LR881103.1	NZ_CP035214.1	16548	5914592	4675	12126	16499	2378985	2374312	2371719	2390239
64	LR881103.1	NC_015968.1	16548	4812833	3107	12229	15327	4260640	4257536	4251287	4268785
64	LR881103.1	NZ_CP020388.1	16548	5408082	4327	12237	16548	403350	399027	395976	412314
64	NZ_CP035214.1	NC_015968.1	5914592	4812833	20031	2406622	2426580	4291618	4311590	-	-
64	NZ_CP035214.1	NZ_CP020388.1	5914592	5408082	28563	2428090	2456422	1176073	1147748	-	-
64	NC_015968.1	NZ_CP020388.1	4812833	5408082	14784	4296848	4311604	1192305	1177552	-	-

Continued on next page

Table 10 – continued from previous page

CI	Q Species	S Species	Q length	S length	A Len	Q Start	Q End	S Start	S End	GI Start	GI End
73	MK422452.1	NZ_LT556084.1	34141	5048670	6253	16852	23087	2178196	2184423	2174695	2191206
92	NC_004313.1	NZ_CP030787.1	40149	4659718	7813	1	7781	1010446	1018233	994657	1036029
94	NC_021857.1	NZ_CP030787.1	41475	4659718	7899	1	7898	1010336	1018233	994657	1036029
96	NC_022749.1	NZ_CP030787.1	39758	4659718	6741	1	6731	1010394	1017125	994657	1036029
200	MF417956.1	NZ_CP035214.1	16570	5914592	3271	12052	15315	2383552	2380297	2371719	2390239
200	MF417956.1	NC_015968.1	16570	4812833	3068	12248	15315	4260624	4257557	4251287	4268785
200	MF417956.1	NZ_CP020388.1	16570	5408082	3062	12248	15309	403342	400281	395976	412314
200	NZ_CP035214.1	NC_015968.1	5914592	4812833	20031	2406622	2426580	4291618	4311590	-	-
200	NZ_CP035214.1	NZ_CP020388.1	5914592	5408082	28563	2428090	2456422	1176073	1147748	-	-
200	NC_015968.1	NZ_CP020388.1	4812833	5408082	14784	4296848	4311604	1192305	1177552	-	-
201	MF417957.1	NZ_CP035214.1	16537	5914592	3271	12023	15286	2383552	2380297	2371719	2390239
201	MF417957.1	NC_015968.1	16537	4812833	3068	12219	15286	4260624	4257557	4251287	4268785
201	MF417957.1	NZ_CP020388.1	16537	5408082	3062	12219	15280	403342	400281	395976	412314
201	NZ_CP035214.1	NC_015968.1	5914592	4812833	20031	2406622	2426580	4291618	4311590	-	-
201	NZ_CP035214.1	NZ_CP020388.1	5914592	5408082	28563	2428090	2456422	1176073	1147748	-	-
201	NC_015968.1	NZ_CP020388.1	4812833	5408082	14784	4296848	4311604	1192305	1177552	-	-
383	MK422452.1	NZ_CP037894.1	34141	4820913	5885	16876	22738	2126051	2131911	2107250	2161140
383	MK422452.1	NZ_CP032841.1	34141	4739272	5650	16876	22509	2793064	2787434	2770640	2816045
383	NZ_CP037894.1	NZ_CP032841.1	4820913	4739272	20013	492423	512365	4258623	4238667	-	-
385	MK416021.1	NZ_CP020448.2	38370	5244290	6051	7579	13591	1112570	1106562	1081621	1135661
385	MK416021.1	NZ_CP038469.1	38370	4840504	6053	7579	13591	1215983	1209974	1164030	1236449
385	NZ_CP020448.2	NZ_CP038469.1	5244290	4840504	50359	3935975	3986257	3801923	3852179	-	-
571	MF695815.1	NZ_LS992183.1	39241	5009078	5776	1	5766	4549175	4554940	4546666	4571702
571	MF695815.1	NZ_CP020820.1	39241	4023751	5752	1	5714	1257421	1263131	1234925	1263088
571	NZ_LS992183.1	NZ_CP020820.1	5009078	4023751	8731	490562	499274	457932	466652	-	-
573	MK416014.1	NZ_LS992183.1	29301	5009078	6419	16767	23166	4548538	4554940	4546666	4571702
573	MK416014.1	NZ_CP020820.1	29301	4023751	5853	17304	23114	1257324	1263131	1234925	1263088
573	NZ_LS992183.1	NZ_CP020820.1	5009078	4023751	8731	490562	499274	457932	466652	-	-
1385	NC_019489.1	NZ_CP021927.1	41726	1757573	5574	5658	11201	809268	814806	795677	836587

I MUSCLE Phylogenetic Trees

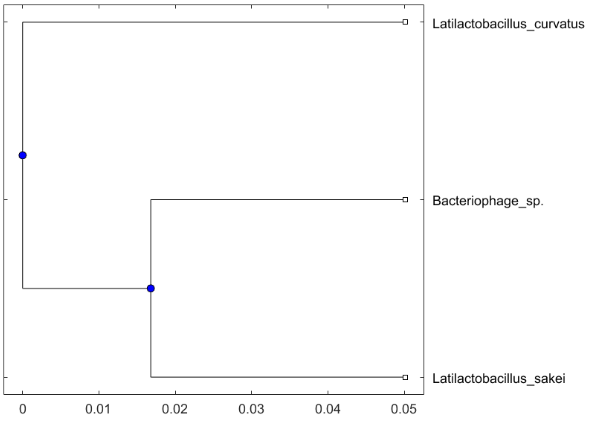

(a) Connection 44

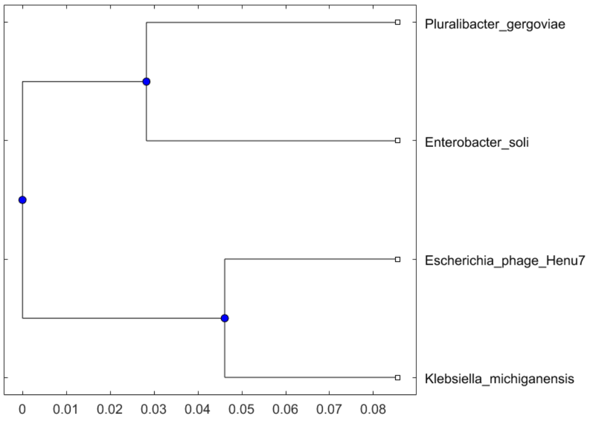

(b) Connection 64

Figure S(7) –The phylogenetic trees of connection 44 and connection 64

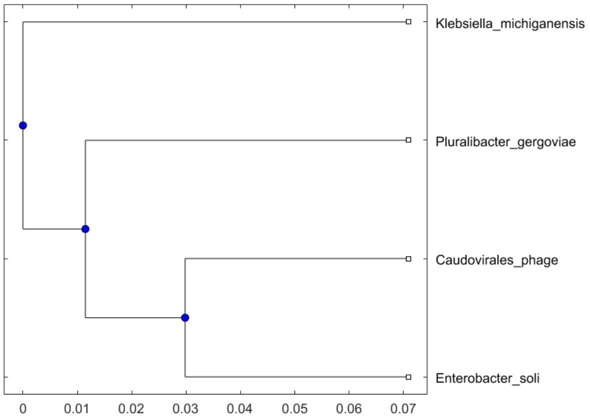

(a) Connection 200

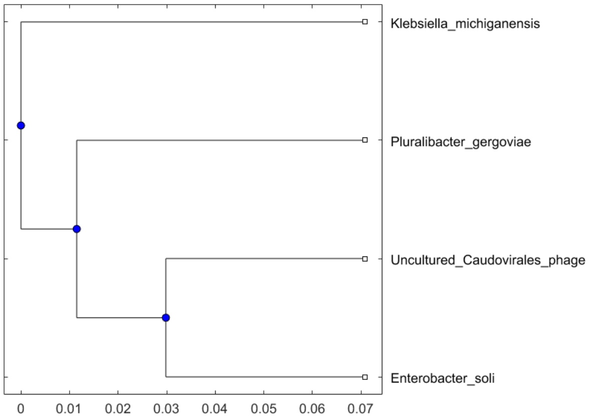

(b) Connection 201

Figure S(8) –The phylogenetic trees of connection 200 and connection 201

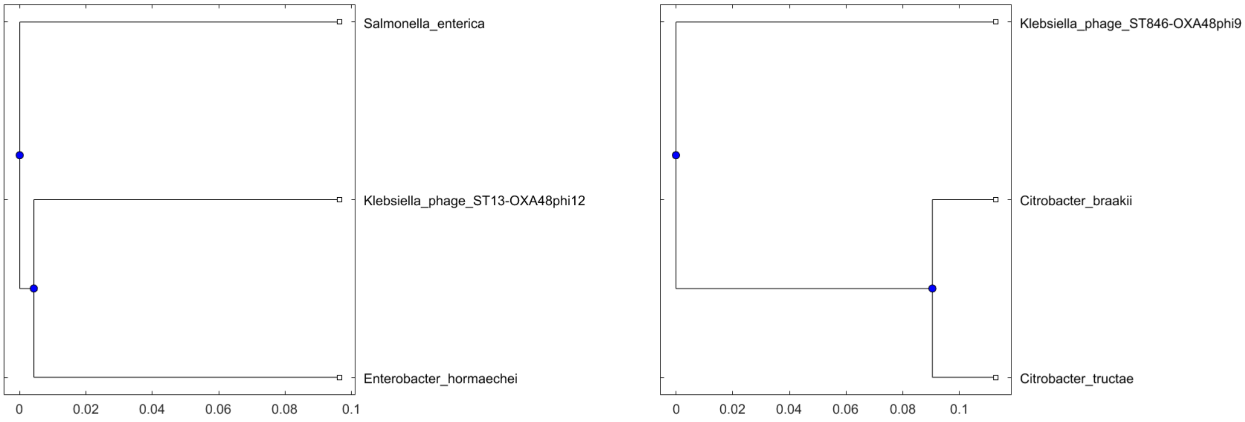

(a) Connection 383 **(b) Connection 385**

Figure S(9) –The phylogenetic trees of connection 383 and connection 385

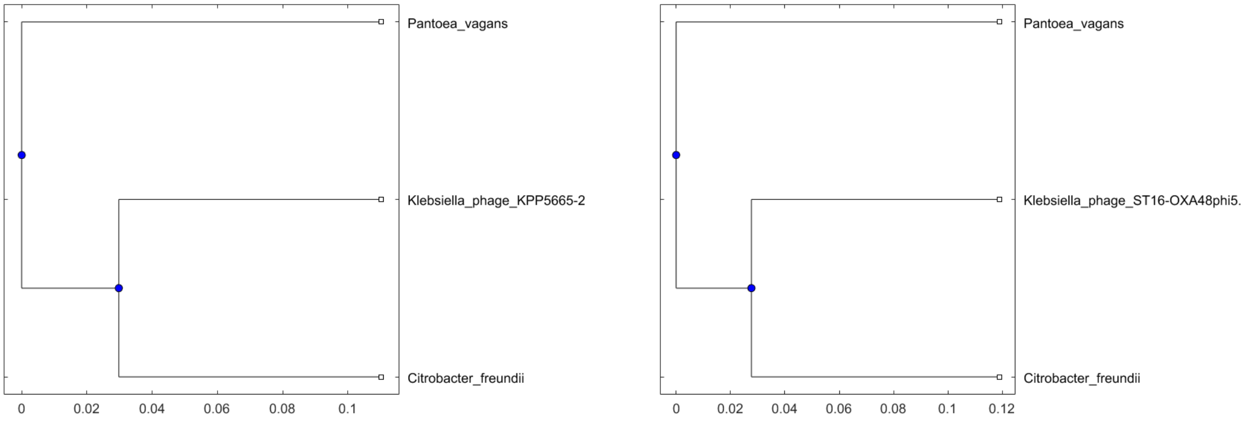

(b) HGT connection 571 **(b) HGT connection 573**

Figure S(10) –The phylogenetic trees of connection 571 and connection 573

References

[1] Ainala, S. K., Seol, E., and Park, S. Complete genome sequence of novel carbon monoxide oxidizing bacteria *Citrobacter amalonaticus* Y19, assembled *de novo*. *Journal of Biotechnology 211* (Oct 2015), 79–80.

[2] Aldahmesh, M. A., Al-Hassnan, Z. N., Aldosari, M., and Alkuraya, F. S. Neuronal ceroid lipofuscinosis caused by MFSD8 mutations: a common theme emerging. *Neurogenetics*

10, 4 (Oct 2009), 307–311.

[3] Alzwghaibi, A. B., Yahyaraeyat, R., Fasaei, B. N., Langeroudi, A. G., and Salehi, T. Z. Rapid molecular identification and differentiation of common *Salmonella* serovars isolated from poultry, domestic animals and foodstuff using multiplex PCR assay. *Archives of Microbiology 200*, 7 (Sep 2018), 1009–1016.

[4] Andino, A., and Hanning, I. *Salmonella enterica*: Survival, Colonization, and Virulence Differences among Serovars. *Scientific World Journal 2015* (Jan. 2015), 520179.

[5] Arenskötter, M., Bröker, D., and Steinbüchel, A. Biology of the metabolically diverse genus *Gordonia*. *Applied and Environmental Microbiology 70*, 6 (Jun 2004), 3195–3204.

[6] Boneca, I. G. The role of peptidoglycan in pathogenesis. *Current Opinion in Microbiology 8*, 1 (Feb 2005), 46–53.

[7] Borenshtein, D., McBee, M. E., and Schauer, D. B. Utility of the *Citrobacter rodentium* infection model in laboratory mice. *Current Opinion in Gastroenterology 24*, 1 (Jan 2008), 32–37.

[8] Bourhy, P., Collet, L., Brisse, S., and Picardeau, M. *Leptospira mayottensis* sp. nov., a pathogenic species of the genus *Leptospira* isolated from humans. *International Journal of Systematic and Evolutionary Microbiology 64*, Pt 12 (Dec 2014), 4061.

[9] Brew, K., Tumbale, P., and Acharya, K. R. Family 6 Glycosyltransferases in Vertebrates and Bacteria: Inactivation and Horizontal Gene Transfer May Enhance Mutualism between Vertebrates and Bacteria. *The Journal of Biological Chemistry 285*, 48 (Nov 2010), 37121.

[10] Brown, S. D., Begemann, M. B., Mormile, M. R., Wall, J. D., Han, C. S., Goodwin, L. A., Pitluck, S., Land, M. L., Hauser, L. J., and Elias, D. A. Complete genome sequence of the haloalkaliphilic, hydrogen-producing bacterium *Halanaerobium hydrogeniformans*. *Journal of Bacteriology 193*, 14 (Jul 2011), 3682–3683.

[11] Cankaya, E., Keles, M., Gulcan, E., Uyanik, A., and Uyanik, H. A Rare Cause of Peritoneal Dialysis-Related Peritonitis: *Achromobacter denitrificans*. *Peritoneal Dialysis International 34*, 1 (Jan 2014), 135.

[12] Challacombe, J., and Kuske, C. Mobile genetic elements in the bacterial phylum Acidobacteria. *Mobile Genetic Elements 2*, 4 (Jul 2012), 179.

[13] Challacombe, J. F., Eichorst, S. A., Hauser, L., Land, M., Xie, G., and Kuske, C. R. Biological Consequences of Ancient Gene Acquisition and Duplication in the Large Genome of *Candidatus* Solibacter usitatus Ellin6076. *PLOS One 6*, 9 (Sep 2011), e24882.

[14] Charlier, D., Piette, J., and Glansdorff, N. IS3 can function as a mobile promoter in *E. coli. Nucleic Acids Research 10*, 19 (Oct 1982), 5935.

[15] Coenye, T., Vancanneyt, M., Cnockaert, M. C., Falsen, E., Swings, J., and Vandamme, P. *Kerstersia gyiorum* gen. nov., sp. nov., a novel *Alcaligenes faecalis*-like organism isolated from human clinical samples, and reclassification of *Alcaligenes denitrificans* Rüger and Tan 1983 as *Achromobacter denitrificans* comb. nov. *International Journal of Systematic and Evolutionary Microbiology 53*, 6 (Nov 2003), 1825–1831.

[16] Cordonin, C., Turpin, M., Bringart, M., Bascands, J.-L., Flores, O., Dellagi, K., Mavingui, P., Roche, M., and Tortosa, P. Pathogenic *Leptospira* and their animal reservoirs: testing host specificity through experimental infection. *Scientific Reports 10*, 7239 (Apr 2020), 1–8.

[17] Davidson, A. L., Dassa, E., Orelle, C., and Chen, J. Structure, function, and evolution of bacterial ATP-binding cassette systems. *Microbiology and Molecular Biology Reviews 72*, 2 (Jun 2008), 317–364.

[18] Davin-Regli, A., Bosi, C., Charrel, R., Ageron, E., Papazian, L., Grimont, P. A., Cremieux, A., and Bollet, C. A nosocomial outbreak due to Enterobacter cloacae strains with the E. hormaechei genotype in patients treated with fluoroquinolones. *Journal of Clinical Microbiology 35*, 4 (Apr 1997), 1008–1010.

[19] Ehrich, S., Behrens, D., Lebedeva, E., Ludwig, W., and Bock, E. A new obligately chemolithoautotrophic, nitrite-oxidizing bacterium,*Nitrospira moscoviensis* sp. nov. and its phylogenetic relationship. *Archives of Microbiology 164*, 1 (Jul 1995), 16–23.

[20] Gaillot, O., di Camillo, P., Berche, P., Courcol, R., and Savage, C. Comparison of CHROMagar Salmonella medium and hektoen enteric agar for isolation of salmonellae from stool samples. *Journal of Clinical Microbiology 37*, 3 (Mar 1999), 762–765.

[21] Gfeller, K. Y. *Molecular analysis of antimicrobial resistance determinants of commensal lactobacilli.* PhD thesis, s.n., Huelva, Spain, 2003.

[22] Hania, W. B., Joseph, M., Bunk, B., Spröer, C., Klenk, H.-P., Fardeau, M.-L., and Spring, S. Characterization of the first cultured representative of a *Bacteroidetes* clade specialized on the scavenging of cyanobacteria. *Environmental Microbiology 19*, 3 (Mar 2017), 1134–1148.

[23] I.B., H., S.P.C., C., K., K., and C.F., H. *ABC proteins: from bacteria to man.* Academic Press, London, UK, 2003.

[24] Khalifa, H. O., Oreiby, A. F., El-Hafeez, A. A. A., Okanda, T., Haque, A., Anwar, K. S., Tanaka, M., Miyako, K., Tsuji, S., Kato, Y., and Matsumoto, T. First Report of Multidrug-Resistant Carbapenemase-Producing Bacteria Coharboring *mcr-9* Associated with Respiratory Disease Complex in Pets: Potential of Animal-Human Transmission. *Antimicrobial Agents and Chemotherapy 65*, 1 (Dec 2020), 01890–01820.

[25] Kiss, H., Lang, E., Lapidus, A., Copeland, A., Nolan, M., Del Rio, T. G., Chen, F., Lucas, S., Tice, H., Cheng, J.-F., Han, C., Goodwin, L., Pitluck, S., Liolios, K., Pati, A., Ivanova, N., Mavromatis, K., Chen, A., Palaniappan, K., Land, M., Hauser, L., Chang, Y.-J., Jeffries, C. D., Detter, J. C., Brettin, T., Spring, S., Rohde, M., Göker, M., Woyke, T., Bristow, J., Eisen, J. A., Markowitz, V., Hugenholtz, P., Kyrpides, N. C., and Klenk, H.-P. Complete genome sequence of *Denitrovibrio acetiphilus* type strain (N2460T). *Standards in Genomic Sciences 2*, 3 (Jun 2010), 270.

[26] Lipsky, B. A., Hook, E. W., Smith, A. A., and Plorde, J. J. Citrobacter Infections in Humans: Experience at the Seattle Veterans Administration Medical Center and a Review of the Literature. *Reviews of Infectious Diseases 2*, 5 (Sep 1980), 746–760.

[27] Macheboeuf, P., Contreras-Martel, C., Job, V., Dideberg, O., and Dessen, A. Penicillin Binding Proteins: key players in bacterial cell cycle and drug resistance processes. *FEMS Microbiology Reviews 30*, 5 (Sep 2006), 673–691.

[28] Maertens, G. N., Engelman, A. N., and Cherepanov, P. Structure and function of retroviral integrase - Nature Reviews Microbiology. *Nature Reviews Microbiology 20* (Jan 2022), 20–34.

[29] Marger, M. D., and Saier, Jr., M. H. A major superfamily of transmembrane facilitators that catalyse uniport, symport and antiport. *Trends in Biochemical Sciences 18*, 1 (Jan 1993), 13–20.

[30] Milton, M. E., Minrovic, B. M., Harris, D. L., Kang, B., Jung, D., Lewis, C. P., Thompson, R. J., Melander, R. J., Zeng, D., Melander, C., and Cavanagh, J. Re-sensitizing Multidrug Resistant Bacteria to Antibiotics by Targeting Bacterial Response Regulators: Characterization and Comparison of Interactions between 2-Aminoimidazoles and the Response Regulators BfmR from *Acinetobacter baumannii* and QseB from *Francisella* spp. *Frontiers in Molecular Biosciences 5*, 15 (Feb 2018), 12 pages.

[31] Mizuno, C. M., Luong, T., Cederstrom, R., Krupovic, M., Debarbieux, L., and Roach, D. R. Isolation and Characterization of Bacteriophages That Infect *Citrobacter rodentium*, a Model Pathogen for Intestinal Diseases. *Viruses 12*, 7 (Jul 2020), 737.

[32] Myhr, S., and Torsvik, T. *Denitrovibrio acetiphilus*, a novel genus and species of dissimilatory nitrate-reducing bacterium isolated from an oil reservoir model column. *International Journal of Systematic and Evolutionary Microbiology 50*, 4 (Jul 2000), 1611–1619.

[33] Nicolas, E., Lambin, M., Dandoy, D., Galloy, C., Nguyen, N., Oger, C. A., and Hallet, B. The Tn3-family of Replicative Transposons. *Microbiology Spectrum 3*, 4 (Aug 2015), 3.4.14.

[34] O'Hara, C. M., Steigerwalt, A. G., Hill, B. C., J J. Farmer, r., Fanning, G. R., and Brenner, D. J. *Enterobacter hormaechei*, a new species of the family *Enterobacteriaceae* formerly known as enteric group 75. *Journal of Clinical Microbiology 27*, 9 (Sep 1989), 2046.

[35] Pascual, J. M., Wang, D., Lecumberri, B., Yang, H., Mao, X., Yang, R., and De Vivo, D. C. GLUT1 deficiency and other glucose transporter diseases. *European Journal of Endocrinology 150*, 5 (May 2004), 627–633.

[36] Pilar, A. V. C., Petronella, N., Dussault, F. M., Verster, A. J., Bekal, S., Levesque, R. C., Goodridge, L., and Tamber, S. Similar yet different: phylogenomic analysis to delineate *Salmonella* and *Citrobacter* species boundaries. *BMC Genomics 21*, 1 (Dec 2020), 1–13.

[37] Prelić, A., Bleuler, S., Zimmermann, P., Wille, A., Bühlmann, P., Gruissem, W., Hennig, L., Thiele, L., and Zitzler, E. A systematic comparison and evaluation of biclustering methods for gene expression data. *Bioinformatics 22*, 9 (May 2006), 1122–1129.

[38] Quirk, P. G., Guffanti, A. A., Clejan, S., Cheng, J., and Krulwich, T. A. Isolation of Tn*917* insertional mutants of *Bacillus subtilis* that are resistant to the protonophore carbonyl cyanide m-chlorophenylhydrazone. *Biochimica et Biophysica Acta 1186*, 1-2 (Jun 1994), 27–34.

[39] Shen, F.-T., Goodfellow, M., Jones, A. L., Chen, Y.-P., Arun, A. B., Lai, W.-A., Rekha, P. D., and Young, C.-C. *Gordonia soli* sp. nov., a novel actinomycete isolated from soil. *International Journal of Systematic and Evolutionary Microbiology 56*, 11 (Nov 2006), 2597–2601.

[40] Søgaard-Andersen, L. Discovery of a Diverse Set of Bacteria That Build Their Cell Walls without the Canonical Peptidoglycan Polymerase aPBP. *mBio 12*, 4 (Jul 2021), e01342–21.

[41] Solinas, F., Marconi, A. M., Ruzzi, M., and Zennaro, E. Characterization and sequence of a novel insertion sequence, IS*162*, from *Pseudomonas fluorescens*. *Gene 155*, 1 (Mar 1995), 77–82.

[42] Tam, R., and Saier, Jr., M. H. Structural, functional, and evolutionary relationships among extracellular solute-binding receptors of bacteria. *Microbiological Reviews 57*, 2 (Jun 1993), 320–346.

[43] Tian, J., Wang, L., Liu, P., Geng, Y., Zhu, G., Zheng, R., Liu, Z., Zhao, Y., Yang, J., and Peng, F. *Deinococcus psychrotolerans* sp. nov., isolated from soil on the South Shetland Islands, Antarctica. *International Journal of Systematic and Evolutionary Microbiology 69*, 12 (Dec 2019), 3696–3701.

[44] Wright, S. N., Gerry, J. S., Busowski, M. T., Klochko, A. Y., McNulty, S. G., Brown, S. A., Sieger, B. E., Michaels, P. K., and Wallace, M. R. *Gordonia bronchialis* sternal wound infection in 3 patients following open heart surgery: intraoperative transmission from a healthcare worker. *Infection Control and Hospital Epidemiology 33*, 12 (Dec 2012), 1238–1241.

[45] Yeo, C. C., and Poh, C. L. Characterization of IS*1474*, an insertion sequence of the IS*21* family isolated from *Pseudomonas alcaligenes* NCIB 9867. *FEMS Microbiology Letters 149*, 2 (Apr 1997), 257–263.

[46] Zehr, J. P., Bench, S. R., Carter, B. J., Hewson, I., Niazi, F., Shi, T., Tripp, H. J., and Affourtit, J. P. Globally Distributed Uncultivated Oceanic N_2-Fixing Cyanobacteria Lack Oxygenic Photosystem II. *Science 322*, 5904 (Nov 2008), 1110–1112.

[47] Zuegg, J., Muldoon, C., Adamson, G., McKeveney, D., Le Thanh, G., Premraj, R., Becker, B., Cheng, M., Elliott, A. G., Huang, J. X., Butler, M. S., Bajaj, M., Seifert, J., Singh, L., Galley, N. F., Roper, D. I., Lloyd, A. J., Dowson, C. G., Cheng, T.-J., Cheng, W.-C., Demon, D., Meyer, E., Meutermans, W., and Cooper, M. A. Carbohydrate scaffolds as glycosyltransferase inhibitors with *in vivo* antibacterial activity. *Nature Communications 6*, 7719 (Jul 2015), 1–11.
